# Metabolism and Effects on Endogenous Metabolism of Paracetamol (Acetaminophen) in a Porcine Model of Liver Failure

**DOI:** 10.1093/toxsci/kfaa023

**Published:** 2020-04-06

**Authors:** Rebecca Dargue, Rabiya Zia, Chungho Lau, Andrew W Nicholls, Theo O Dare, Karla Lee, Rajiv Jalan, Muireann Coen, Ian D Wilson

**Affiliations:** k1 Division of Systems Medicine, Department of Metabolism, Digestion and Reproduction, Imperial College London, South Kensington, London SW7 2AZ, UK; k2 GSK R&D, Ware, Hertfordshire SG12 0DP, UK; k3 Department of Clinical Science and Services, Royal Veterinary College, University of London, Hertfordshire AL9 7TA, UK; k4 UCL Institute for Liver and Digestive Health, Royal Free Hospital, London NW3 2PF, UK; k5 Oncology Safety, Clinical Pharmacology and Safety Sciences, R&D, AstraZeneca, Cambridge, UK

**Keywords:** paracetamol/acetaminophen, metabolism, metabonomics, metabolomics, liver, kidney

## Abstract

The metabolic fate, toxicity, and effects on endogenous metabolism of paracetamol (acetaminophen, APAP) in 22 female Landrace cross large white pigs were evaluated in a model of acute liver failure (ALF). Anesthetized pigs were initially dosed at 250 mg/kg via an oroduodenal tube with APAP serum concentrations maintained above 300 mg/l using maintenance doses of 0.5–4 g/h until ALF. Studies were undertaken to determine both the metabolic fate of APAP and its effects on the endogenous metabolic phenotype of ALF in using ^1^H NMR spectroscopy. Increased concentrations of citrate combined with pre-ALF increases in circulating lactate, pyruvate, and alanine in plasma suggest mitochondrial dysfunction and a switch in hepatic energy metabolism to glycolysis in response to APAP treatment. A specific liquid chromatography-tandem mass spectrometry assay was used to quantify APAP and metabolites. The major circulating and urinary metabolite of APAP was the phenolic glucuronide (APAP-G), followed by *p*-aminophenol glucuronide (PAP-G) formed from N-deacetylated APAP. The PAP produced by N-deacetylation was the likely cause of the methemoglobinemia and kidney toxicity observed in this, and previous, studies in the pig. The phenolic sulfate of APAP, and the glutathione-derived metabolites of the drug were only found as minor components (with the cysteinyl conjugate detected but not the mercapturate). Given its low sulfation, combined with significant capacity for N-deacetylation the pig may represent a poor translational model for toxicology studies for compounds undergoing significant metabolism by sulfation, or which contain amide bonds which when hydrolyzed to unmask an aniline lead to toxicity. However, the pig may provide a useful model where extensive amide hydrolysis is seen for drugs or environmental chemicals in humans, but not in, eg, the rat and dog which are the preclinical species normally employed for safety assessment.

Overdose of the analgesic drug paracetamol (acetaminophen, 4-hydroxyacetanalide, APAP) is a well-known cause of drug-induced liver injury (DILI). As such APAP is a leading cause of acute liver failure (ALF), which is associated with high mortality and orthotopic liver transplantation (Lee, 2012). A porcine model of ALF has previously been shown to provide a reproducible and clinically relevant model of paracetamol-induced hepatotoxicity (Baker *et al.*, 2015; Lee *et al.*, 2013, 2015) and has been used to test the “University College London Liver Dialysis Device (UCL-LDD)” currently being developed as a treatment for ALF. Previous studies with the model showed that chronic APAP dosing for up to 20 h led to ALF onset (Lee *et al.*, 2013). In humans, and most preclinical species, APAP metabolism occurs primarily in the liver. The major biotransformations involve conjugation to form the phenolic glucuronide (around 50%) and sulfate conjugates (30%–40%) (Mazaleuskaya *et al.*, 2015) although in rat the major metabolite is the sulfate (McGill *et al.*, 2012). With increasing dose the formation of the sulfate plateaus, and oxidative metabolism mediated by cytochrome P450 increases, leading to the formation of the toxic reactive intermediate N-acetyl-*p*-benzoquinoneimine (NAPQI). NAPQI is detoxified by reaction with glutathione (GSH) to form the glutathionyl conjugate, which is further metabolized in the kidney to form the N-acetylcysteinyl conjugate, concentrations of which increase with dose (Mazaleuskaya *et al.*, 2015). Other extrahepatic metabolism includes conversion of APAP to *p*-aminophenol (PAP) due to deacetylation by carboxylesterases in kidney, which has been observed in eg, the Fischer rat (Newton *et al.*, 1985) as well as cats and dogs (McConkey *et al.*, 2009). PAP formation appears to be a minor route in humans (ca. 1%) and is generally followed by rapid reacetylation (Nicholls *et al.*, 1997). However, little has been reported on the metabolism of APAP in the pig, although it has been shown that pigs have a lower sulfation capacity than humans (Capel *et al.*, 1972). A known consequence of APAP administration to pigs has been the production of methemoglobin (metHb) and to reduce toxicity maintaining serum APAP below 200–300 µg/ml to limit metHb formation has been suggested (He *et al.*, 2017; Lee *et al.*, 2013; Newsome *et al.*, 2010; Thiel *et al.*, 2011). Given the increasing interest in the use of the pig in preclinical toxicity studies there is a clear need to better understand drug metabolism in this species. During the course of this work on the evaluation of efficacy of the UCL-LDD we have taken the opportunity to attempt to better define both the metabolic fate of paracetamol and its effects on the endogenous metabolic phenotype in this porcine model of hepatotoxicity.

## MATERIALS AND METHODS

### 

#### Chemicals and Reagents

Acetaminophen (APAP), its glucuronide (APAP-G) conjugate (sodium salt), and APAP-d3 were purchased from Sigma Aldrich (Gillingham, UK), its sulfate (APAP-S) (potassium salt), cysteinyl (APAP-C) (trifluoroacetic acid salt, TFA), N-acetylcysteinyl (APAP-NAC) (disodium salt), glutathione (APAP-SG) (disodium salt), and 3-methoxy (APAP-OMe) conjugates and the deuterated internal standards, APAP-S-d3 (potassium salt), APAP-G-d3 (sodium salt), APAP-C-d5 (TFA salt), APAP-NAC-d5 (sodium salt), and APAP-SG-d3 (disodium salt), were from Toronto Research Chemicals (Toronto, Canada). *p*-Aminophenol-glucuronide (PAP-G) was from Santa Cruz Biotechnology (Dallas, Texas). Optima grade water was from Fisher Scientific (Leicester, UK), LC-MS grade solvents and formic acid (FA) were from Sigma Aldrich (Poole, UK).

#### Animal Model and Study Design

The study methodology has been described elsewhere (Lee *et al.*, 2015). Briefly, 22 female Landrace cross Large White Pigs (26–36 kg), between 4 and 6 weeks of age, were randomized into 3 groups: APAP plus UCL-LDD (APAP-UCL-LDD; *n *=* *9), APAP plus Control Device (APAP-CD; *n *=* *9), or control plus CD (control-CD; *n *=* *4). Animals were maintained under total intravenous anesthesia with intermittent positive pressure ventilation and intensive care monitoring and management. To induce ALF, APAP (initially 250 mg/kg as an aqueous suspension) was administered via an oroduodenal tube, APAP serum concentrations were maintained above 300 mg/l with maintenance APAP doses of 0.5–4 g hourly. Dosing continued until the prothrombin time (used as a clinical marker of liver dysfunction) as defined by an international normalized ratio (Kirkwood, 1983) became > 3 at which point the animal was considered to have reach ALF. Two hours after onset of ALF, treatment with either the UCL-LDD or a control device (CD) was initiated until death. “Death” was defined as nonrecoverable cardiorespiratory arrest or when 2 of the following criteria were reached: hematocrit < 10%; blood potassium > 5.5 mmol/l; blood lactate > 10 mmol/l; blood pH < 7.25; partial pressure of oxygen in arterial blood (P_a_O_2_) <60 mmHg. An albumin infusion protocol was developed based on a pilot study (Lee *et al.*, 2013). At the onset of APAP dosing 20% human serum albumin was infused at 1.6 g albumin/h increasing to 16 g/h at 12 h of APAP dosing finally increasing to 20 g/h at ALF. Control animals were infused with 1.6 g albumin/h throughout the study. Albumin infusion was stopped if the serum albumin concentration was greater than 20 g/l. 2 Units of fresh frozen porcine plasma were given at ALF in both groups. All animals were maintained throughout the study with an IV infusion of “Viamin 14” containing a number of amino acids (Supplementary Information 1). In addition, intravenous fluid therapy to support critically ill patients, containing sodium lactate, 0.9% sodium chloride, 8.4% sodium bicarbonate, 6% hydroxyethyl starch 130/0.4, and potassium chloride, was administered according to set protocols. Serum and plasma sampling was conducted every 4 h and the following parameters were measured in serum: Serum urea (mmol/l), creatinine (µmol/l), total bilirubin (µmol/l), AST (U/l), ALP (U/l), albumin (g/l). Arterial blood gas monitoring pre- and post-ALF included lactate mmol/l and % metHb and were determined hourly (Supplementary Table 1) as was serum paracetamol (mg/l) (see Lee *et al.*, 2015). Anesthetic monitor data were taken every 15 min, hematology was taken every 4 h. Liver biopsies were taken at time 0, 12 h, and study endpoint. Kidney samples were taken at study endpoint, with histopathology performed on both tissues. Urine samples were taken at 4-h intervals throughout the study. Full methods are provided in previous reports (Baker *et al.*, 2015; Lee *et al.*, 2013, 2015). The study design is summarized in Supplementary Figure 1. All animal procedures complied with the UK “Animals (Scientific Procedures) Act 1986.”

#### ^1^H NMR Spectroscopy of Aqueous Tissue Extracts and Urine

##### ^1^H NMR spectroscopy of aqueous renal extracts

Kidney extractions for ^1^H NMR spectroscopic analysis were performed as described elsewhere (Beckonert *et al.*, 2007; Want *et al.*, 2013). Briefly, 1.5 ml ice-cold methanol/water (1:1, v/v) and kidney tissue (mean weight 68.5 ± 6.7 mg) were placed into bead beater tubes. Samples were homogenized using a bead beater (Precylls24, Bertin) using 2 cycles of 6500 Hz for 40 s with samples cooled on dry-ice in-between cycles. Samples were then centrifuged at 10 000 × g for 10 min (4°C) with 1 ml of supernatant transferred to Eppendorf tubes for concentration in a centrifugal evaporator (Eppendorf Concentrator Plus) (45°C overnight). Dried supernatants were reconstituted in 580 μl pH 7.4 tissue buffer (Supplementary Information 2) prepared in D_2_O containing 0.5 mM sodium 3-(trimethylsilyl)-[2,2,3,3-2H4]-propionic acid (TSP), vortexed for 90 s and centrifuged at 12 000 × g for (5 min 4°C). (This buffer was also used for all sample types analyzed by ^1^H NMR spectroscopy.) Supernatants (550 μl) were transferred to 5 mm NMR tubes (HP-507, Norell) which were inserted into an automated sample handling carousel (Bruker Biospin, Germany).

##### ^1^H NMR spectroscopy of hepatic aqueous extracts

Liver extracts were prepared using the approach used for kidney except that the volume of tissue buffer (Supplementary Information 2) was adjusted (20:1, V/W) to the tissue weight (mean 50.6 ± 7.6 mg).

##### ^1^H NMR spectroscopy of urine samples

Urine samples were prepared as described elsewhere (Dona *et al.*, 2014). Briefly, 600 μl urine was centrifuged at 12 000 × g for 5 min at 4°C. Supernatants (540 μl) were transferred to 5 mm NMR tubes containing urine buffer (60 μl, Supplementary Information 2). ^1^H NMR spectra for urine and tissue extracts were acquired on a Bruker Avance-600 spectrometer, using a 4 mm BBI probe (Bruker), operating at 600.1 MHz ^1^H frequency and at a temperature of 300 K. A standard one-dimensional solvent suppression pulse sequence was used to acquire the free induction decay (FID; relaxation delay-90° pulse-4 μs delay-90° pulse-mixing time-90° pulse-acquire FID). The D_2_O and TSP present in the buffer provided a field frequency lock and chemical shift reference compound (*δ*^1^H = 0.00), respectively. For liver extracts and urine 128 transients were collected whilst for kidney extracts the number was increased to 256. The data from all of these experiments were collected into 65 000 data points using a spectral width of 12 019.2 Hz, relaxation delay of 4 s and an acquisition time of 2.73 s.

##### ^1^H NMR spectroscopy of plasma samples

Plasma samples were prepared as described elsewhere (Dona *et al.*, 2014). Spectral data were acquired using a Carr-Purcell-Meiboom-Gill pulse sequence (Beckonert *et al.*, 2007). Thirty-two scans were collected into 64 K data points, using a spectral width of 12 019.23 Hz. Suppression of the water signal was performed during the relaxation delay (d1) of 4 s, acquisition time was 3.07 s. The D_2_O present in the buffer provided a field frequency lock.

##### ^1^H NMR spectral data processing

All ^1^H NMR spectra were initially processed in TopSpin (v3.1, Bruker), where a line-broadening factor of 0.3 Hz was applied prior to Fourier transformation. Spectra were automatically phased, baseline-corrected and referenced to TSP (*δ* = 0.00). Using an in-house script, ^1^H NMR data were imported to MATLAB (R2016b, MathWorks). TSP and water resonances were removed, before applying probabilistic quotient normalization for urine and tissue spectra (Dieterle *et al.*, 2006). For urine and plasma spectra, peaks were manually aligned using in-house scripts (Veselkov *et al.*, 2009) before normalization. Endogenous metabolite assignments were performed using Statistical TOtal Correlation Spectroscopy (STOCSY) (Cloarec *et al.*, 2005), J-resolved Spectroscopy (JRES), spectral databases and literature (Kyriakides *et al*., 2016; Merrifield *et al.*, 2011; Nicholson *et al.*, 2002). APAP metabolites were assigned using STOCSY, JRES, literature (Bales *et al.*, 1988; Gartland *et al.*, 1990; Kyriakides *et al.*, 2016) and standards.

##### Univariate statistical analysis of ^1^H NMR data

Prism 8.0 (GraphPad, La Jolla, California) was used for univariate analysis of integrated ^1^H NMR spectral resonances. Kruskal-Wallis with multiple comparison with a FDR multiple test correction (2-stage linear step-up procedure of Benjamini, Krieger, and Yekutieli) was used to compare among 3 or more groups and Mann-Whitney was used for comparison between 2 groups. Initially statistical comparisons were made among the 3 groups at each time point for Control, APAP+CD, and APAP + UCL-LDD. As there was no statistical significance between the 2 device groups for any of the metabolites they were combined for further analysis.

#### UPLC-MS/MS Analysis of Plasma

##### Sample preparation and analysis

Standard curves, QC, and plasma samples were prepared as described in Supplementary Information 3, Tables 6–12, and Figure 10. Briefly, 5 μl of plasma were diluted with 85 μl of MeOH and 10 μl of internal standard stock solution added. Samples were kept at −20°C for 20 min to precipitate proteins, centrifuged (10 min, 10 000 × g) and 20 μl of supernatant diluted with water (980 μl) for analysis.

##### Chromatography

Analysis was on an Acquity UPLC system using a 2.1 i.d. X 100 mm Acquity HSS T3 column packed with a 1.8 µm 130A C18 stationary phase (Waters Corporation) using the run order sequences shown in Supplementary Figure 11. The separation was performed at 40°C, at a flow rate of 0.6 ml/min, using a reversed-phase gradient, with water: 0.1% (v/v) FA as solvent A and methanol:0.1% (v/v) FA as solvent B. The gradient starting conditions were 5% solvent B for 0.5 min, going then, in a series of linear gradients to 7% B (1.85 min) then 8% (1.9 min) 10% (2.5 min), 16% (4.0 min), 25% (5 min), and finally to 95% (5.1 min) for column washing. The wash solvent conditions were maintained at 95% B for 0.9 min, returning to the starting conditions by 6.1 min. With a reequilibration time of 1.4 min the overall analysis time was 7.5 min/sample (the separation is illustrated in Supplementary Figures 12 and 13).

##### Mass spectrometry

MS/MS Data were acquired using a Waters Xevo tandem quadrupole (TQ)-S mass spectrometer (Waters Corporation, Manchester, UK) operating in positive ion electrospray (ESI) mode. MS conditions are given in Supplementary Table 13 (together with analyte retention time data). The capillary voltage was set to 3 kV, the source temperature was 150°C, the desolvation temperature was 500°C, and the gas flow 1000 l/h. Cone voltages and collision energies were optimized for each compound.

##### LCMS/MS data analysis

Raw LC-MS data were processed by the TargetLynx application package within MassLynx software (Waters Corporation). The raw data were mean smoothed and peak integration was performed using the ApexTrack algorithm. Concentrations of APAP and metabolites were determined by reference to standard curves.

## RESULTS

### 

#### Survival, Histopathological, and Biochemical Characterization of the ALF Model

**Figure 1.**
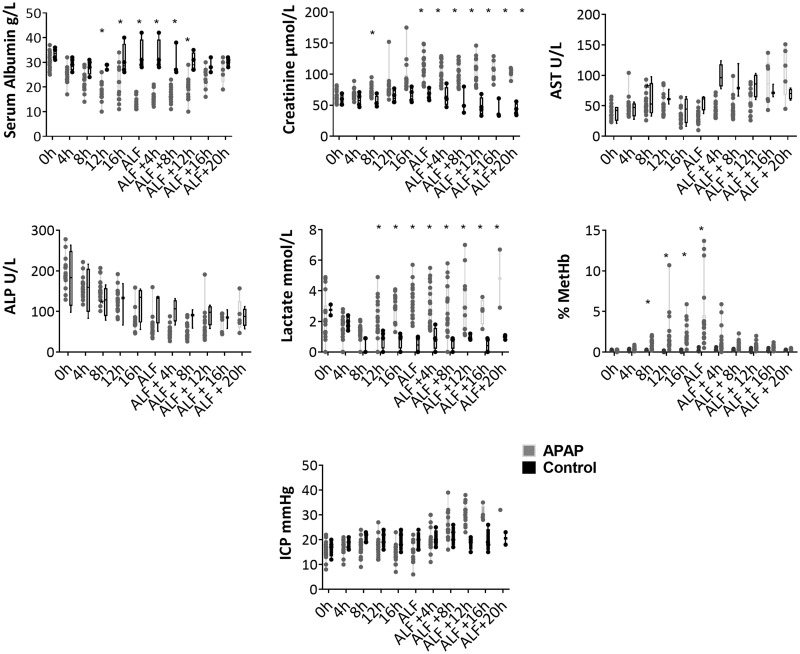
Parameters measured to characterize the ALF model. Each is plotted at 4-h intervals up to ALF, and thereafter at 4-h intervals to postmortem. The figure shows, from left to right, upper row; albumin, creatinine, and AST. Middle row; ALP, blood lactate, and blood % metHb and bottom row; ICP measured via anesthetic monitoring. Boxes display median and interquartile range and the whiskers show the full range. Asterisk (*) indicates where APAP-treated animals were significantly different from controls with a *q* value <.05 after FDR correction, following Kruskal-Wallis with multiple comparisons.

Following the administration of APAP, ALF was recorded at a median time of 17.5 ± 2.7 h (range: 12–23 h) with a median total administered dose of 48.5 g (31.5–68.5 g). Median survival time from ALF for APAP-treated animals was 13 ± 3.5 h, with a range from 8 to 20 h (all surviving animals were terminated at 20-h post-ALF). Postmortem histopathological analysis of liver specimens showed acute centrilobular to midzonal hepatocyte degeneration and necrosis in all APAP-treated animals, but not in control animals (see Supplementary Figure 2). Postmortem histopathological analysis of renal specimens showed tubular epithelial cell swelling and protein loss in all APAP-treated animals, but not in control animals. At the onset of ALF serum creatinine concentrations were significantly higher in APAP-treated animals than the controls, whereas serum albumin concentrations were significantly lower (Figure 1). APAP-treated animals showed hematotoxicity, with significantly increased blood concentrations of methemoglobin and lactate (Figure 1) and significant increases in prothrombin time (21 ± 1.3 s at 0 h, 80 ± 19 s at ALF). MetHb concentrations increased with APAP dosing but decreased upon cessation of dosing at the onset of ALF (Figure 1) and were highly variable, ranging from 0.5% to 13.7% at that point. Intracranial pressure (ICP) increased in APAP-treated animals over the course of the experiment, whereas in control animals it remained more constant, although the difference between treated and control groups was not statistically significant (Figure 1). ALP, AST, which has a high sensitivity for hepatocyte injury in the pig, unlike ALT which does not increase significantly in this model, (Lee *et al.*, 2013) (Figure 1), bilirubin, and urea concentrations were also measured with median and interquartile range for each time point provided in Supplementary Table 1.

#### ^1^H NMR Spectroscopy of Tissue Extracts and Biofluids

**Figure 2.**
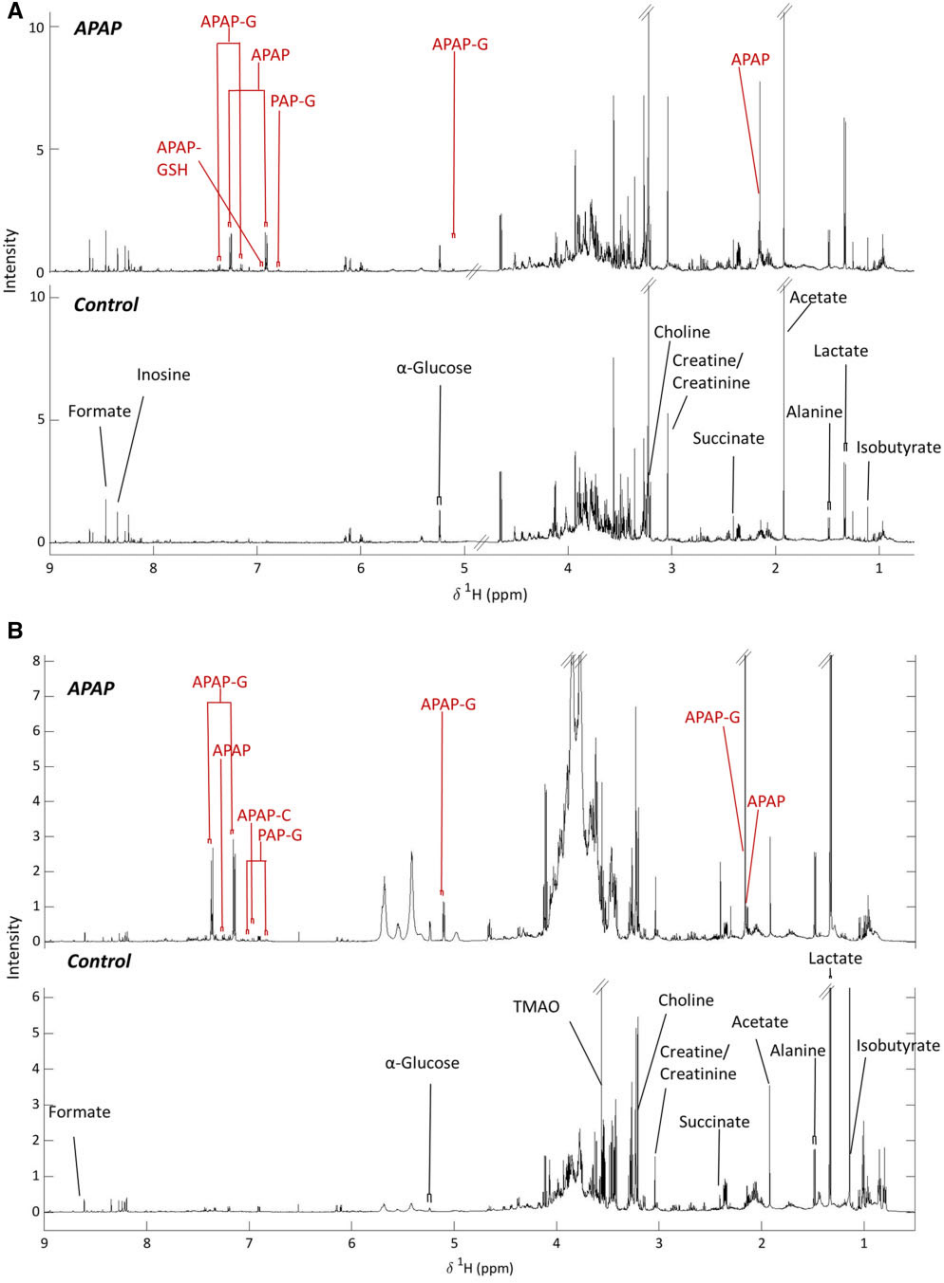
Representative ^1^H NMR spectra of porcine liver (UPPER) (at ALF in an APAP treated and a Control animal at 20 h) and kidney (LOWER) at postmortem with key endogenous and drug metabolite resonances labeled.

Representative ^1^H NMR spectra for both liver and kidney tissue extracts for control and APAP-treated animals (12-h post-APAP administration) are shown in Figure 2 whilst Supplementary Figures 3 and 4 show the equivalent results for plasma and urine, respectively. The ^1^H NMR spectra from all matrices, but kidney extracts and urine in particular, showed convoluted signals between 3 and 4 ppm, from overlapped sugar and amino acid resonances, which prevented the integration of peaks in this region. Analysis of the data obtained by ^1^H NMR spectroscopy showed that, there were no statistically significant differences between the metabolite profiles of the APAP+CD and APAP + UCL-LDD animals, for any of the detected metabolites, and therefore (as discussed in the Materials and Methods section) their results were combined.

#### Plasma

In the ^1^H NMR spectra generated from plasma samples 18 endogenous metabolites were identified including valine, isobutyrate, alanine, lysine, acetate, pyruvate, glutamine, citrate, proline, lactate, glucose, histidine, tyrosine, phenylalanine, and formate (Supplementary Figures 3 and 5). In addition overlapped resonances for creatine and creatinine were also noted (Supplementary Figures 3 and 5). Following up to 8 h of APAP treatment glutamine was significantly decreased in concentration in the plasma of drug-treated animals, whereas tyrosine concentrations were increased. From 16-h postcommencement of APAP administration the amount of pyruvate detected in plasma was significantly increased in drug-treated animals whilst plasma glucose and lactate concentrations both increased significantly from the onset of ALF. Citrate concentrations were increased in APAP-treated animals compared with controls from 4 h after the onset of ALF whilst glutamine concentrations increased in the later stages of ALF (Table 1 and Supplementary Figure 3).

**Table 1.** Summary of Endogenous Metabolite Changes in APAP-treated Pigs Relative to Controls Across All Matrices at the Pre- and Post-ALF Timepoints

Sample Matrix	Metabolite Changes Pre-ALF	*p* Value	Adjusted *p* Value (*q*)	Metabolite Changes Post-ALF	*p* Value	Adjusted *p* Value (*q*)
Plasma	**Pyruvate**	.0001	.0002	**Pyruvate**	.0005	.0006
	**Lactate**	.002	.0012	**Lactate**	<.0001	.0002
	**Glucose**	.0172	.009	**Glucose**	<.0001	<.0001
	Glutamine	.0002	.0007	**Glutamine**	.0018	.0037
	**Tyrosine**	<.0001	.0002	**Alanine**	.0047	.308
				**Citrate**	<.0001	.0002
Urine	Dimethylamine	.0637	.0418	Dimethylamine	.0012	.0046
	Trimethylamine	<.0001	.0001	Trimethylamine	<.0001	.0001
	Choline	.0025	.0286	**Glucose**	<.0001	.0004
	Creatine	.0003	.0011	Creatine	.0075	.0073
	Creatinine	.0006	.0068			
	**Lactate**	.0001	.0012			
Liver	No significant changes			**Creatine**	<.0001	<.0001
				Isobutyrate	.0117	.0245
				**Succinate**	<.0001	<.0001
				Inosine	.0088	.0184
				**Alanine** ^a^	.0236	.0743
				**Lactate** ^a^	.224	.706
Kidney	No data			**Glucose**	.0003	Not applicable
				**Sucinate**	.0074	
				**Alanine**	.0104	

ALF occurred at 17.5 ± 2.7-h postonset of APAP treatment. Pre-ALF refers to all time points prior to ALF and post-ALF to all time points after ALF. Normal type, metabolite decrease; **Bold**, metabolite increase.

a Changes not statistically significant.

#### Urine

In the ^1^H NMR spectra generated from urine samples (Supplementary Figure 4) 15 endogenous metabolites were identified (Table 1). During APAP treatment, but prior to the onset of ALF, there were significant decreases in the amounts of choline, creatine, and creatinine with concomitant significant increases in lactate concentration. In APAP-treated animals post-ALF, quantities of trimethylamine (TMA) and dimethylamine (DMA) were significantly decreased whilst those of glucose were significantly increased compared with controls. Urinary alanine concentrations were higher following ALF than in controls however, this difference was not statistically significant (Supplementary Figure 6).

#### Aqueous Soluble Tissue Extracts

From the spectra of the hepatic extracts (Figure 2) 15 endogenous metabolites were identified (Supplementary Table 4). Whilst no significant differences were observed at 0  or 12 h in postmortem samples from the controls those of the APAP-treated animals showed increased quantities of succinate, creatine, inosine, and isobutyrate (Table 1 and Supplementary Figure 7). There was also an increase in the amounts of alanine and lactate in liver extracts from APAP-treated animals postmortem, but these were not statistically significant. In the ^1^H NMR spectra of kidney extracts (Figure 2) the amounts of total glucose (α and β), succinate and alanine were significantly increased in APAP-treated animals compared with controls (Table 1 and Supplementary Figure 8).

Changes in the relative amounts of the various endogenous metabolites detected in biofluids or aqueous extracts of organs are summarized in Table 1.

#### APAP Metabolism—^1^H NMR Spectroscopy

##### Plasma

APAP, its glucuronide and PAP-G could be detected in the ^1^H NMR spectra of plasma (Supplementary Figure 3), with APAP-G the most abundant, and present at higher concentrations than APAP throughout most of the experiment. PAP-G was the only other metabolite that could be detected, at lower concentration relative to APAP and APAP-G, at all times.

##### Urine

In urine samples APAP and its glucuronide, sulfate, and cysteinyl conjugates, as well as PAP-G were detected in the ^1^H NMR spectra of urine obtained from dosed, but not control animals. The major metabolite detected was APAP-G, which was present in the largest amounts throughout the experiment, followed by APAP itself, for the first 12 h after the commencement of drug administration, after which PAP-G was seen to provide the second most abundant APAP-related metabolite. Both APAP-S and APAP-Cys, were observed in urine samples, but at relatively low intensities (Supplementary Figure 4).

##### Tissue extracts

In the renal extracts APAP-G was the most abundant metabolite detected, followed by unchanged APAP and APAP-Cys, with PAP-G detected in trace amounts. In the hepatic extracts obtained at 12 h post the start of drug administration, unchanged APAP had the highest abundance, followed by APAP-G and small amounts of APAP-GSH. PAP-G was also present at low intensities (Figure 2 and Supplementary Figure 4).

Supplementary Tables 2–5 provide details of the ^1^H NMR spectral data for each of the endogenous metabolites as well as APAP and the APAP-metabolites detected in the various matrices.

#### U(H)PLC-MS/MS of APAP and Metabolites in Plasma

**Figure 3.**
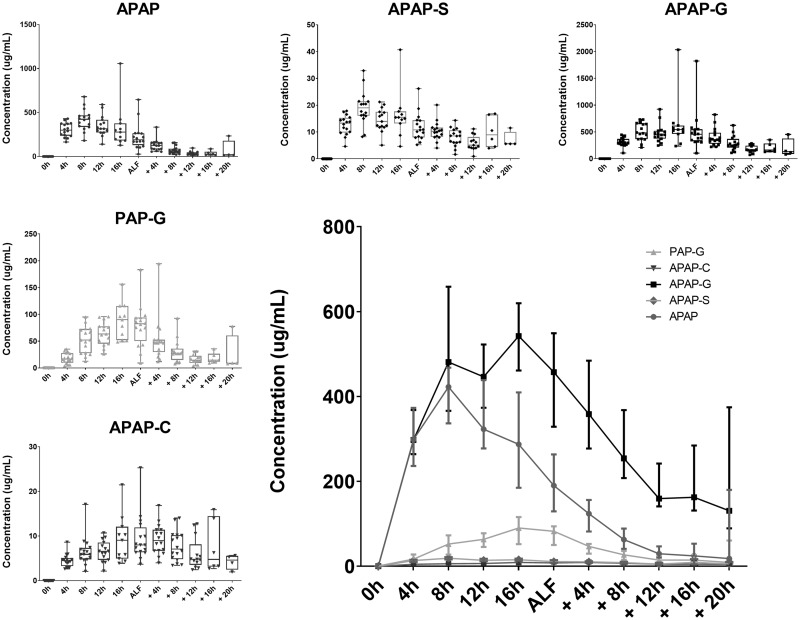
Metabolites quantified in plasma using UHPLC-MS/MS up to, and postanimal-dependent ALF.

APAP and its metabolites were quantified by UPLC-MS/MS using stable isotope-labeled (SIL) internal standards or, in the case of PAP-G where no SIL was available, monitored and semiquantified relative to an external standard curve. This analysis showed that APAP-G was the major APAP-related compound present in the circulation, followed by APAP itself, and then PAP-G. Both APAP-S and APAP-Cys were also detected and quantified but were at relatively low concentrations, whereas APAP-GSH and APAP-NAC were not detected (Figure 3).

## DISCUSSION

### 

#### Paracetamol-induced Changes in Endogenous Metabolite Profiles

**Figure 4.**
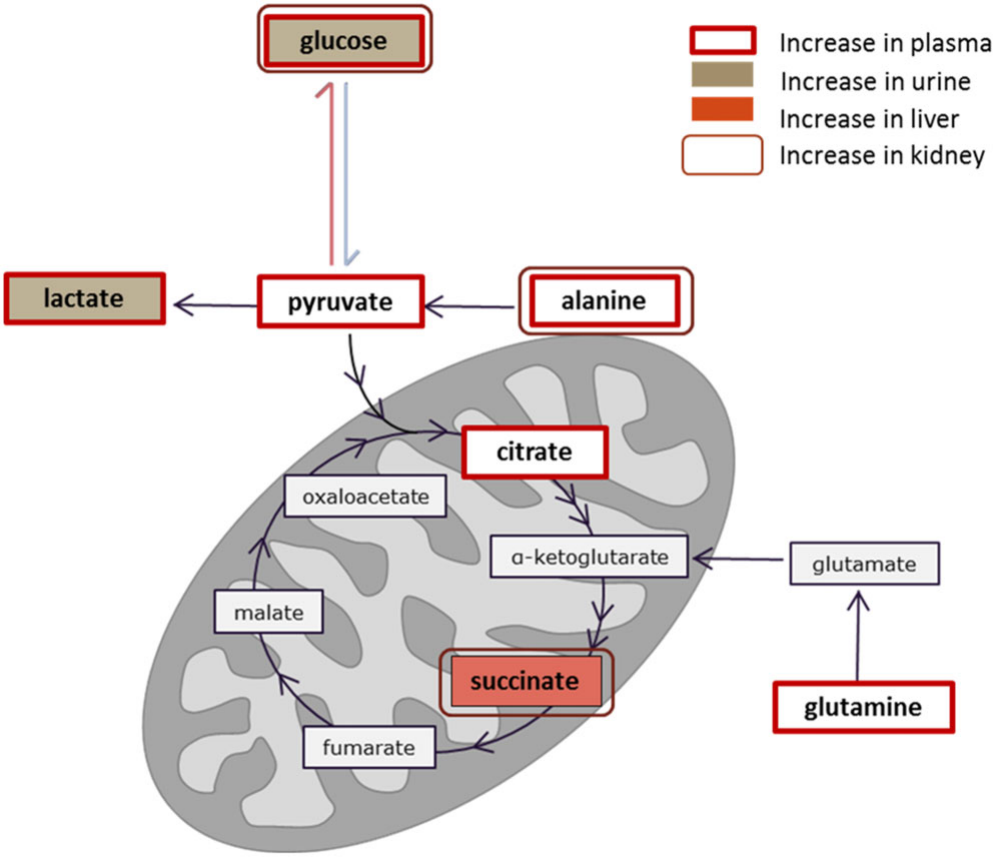
Diagram showing the key components of the TCA cycle within the mitochondria seen to change in 1 or more of the matrices and biofluids investigated. The TCA cycle components have been color coded to show in which of the matrices these components were increased (as per key within figure) in the samples following the onset of ALF.

There were endogenous changes in metabolism which indicated effects on 3 main pathways; energy metabolism, amino acid metabolism, and choline metabolism. Similar changes have been reported previously in APAP-induced ALF with eg, increased concentrations of a number of components of the citric acid cycle, such as succinate (Coen *et al.*, 2003, 2004; Dabos *et al.*, 2011; Kyriakides *et al*., 2016). In the current model there were also increased concentrations of citrate and this, coupled with the pre-ALF increase in circulating lactate, pyruvate, and alanine in plasma indicates that there was a hepatic switch to glycolysis early on after APAP treatment due to mitochondrial dysfunction (see Figure 4). In accordance with previous animals models, but contrary to the report by Dabos *et al.* (2011), we also detected an increase in glucose in the kidney, suggesting gluconeogenesis was upregulated in the kidney to compensate for the reduction in hepatic energy metabolism.

Changes to the circulating glutamine concentrations resemble the glutamine increase seen in human ALF, and this change has been proposed to be linked to hepatic encephalopathy (Rao *et al.*, 2012). The upregulation of glutamine (Holecek, 2014) and urinary decreases in TMA, DMA, and TMAO concentrations in APAP-treated animals may show alteration to gut microbiota (Ussher *et al.*, 2013). Changes to creatine and creatinine in the circulation, urine, and liver have also been previously seen in APAP toxicity and DILI (Kyriakides *et al*., 2016) with an increase in the amounts in the circulation, and decreases in urine, commonly indicative of poor renal clearance. There was also a significant decrease in hepatic inosine concentrations. This may be significant with respect to the methemoglobinemia as porcine erythrocytes use hepatically derived inosine as a substitute for glucose for glucose (Watts *et al.*, 1979) and it has been shown, *in vitro*, that inosine is used to efficiently generate NADH for the enzymatic reduction of methemoglobin in pig erythrocytes (Sartorelli *et al.*, 1996).

#### APAP Metabolism and Its Relation to Toxicity in the Pig

**Figure 5.**
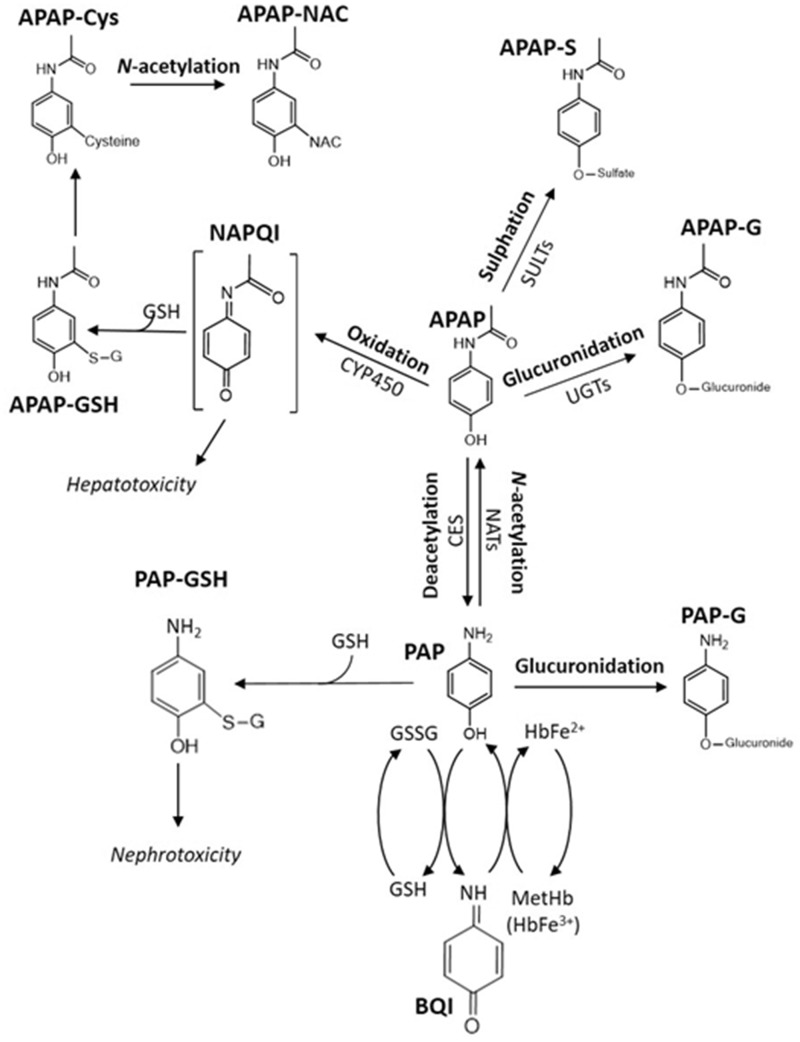
APAP metabolism in the pig showing the formation of PAP, PAP-derived metHb, PAP-G, and the putative nephrotoxin PAP-GSH.

Glucuronidation predominated over sulfation in the metabolism of APAP in the pig with the latter appearing to represent only a minor reaction with low concentrations of the sulfate conjugate detected in urine and the circulation. Similar results were seen for the metabolism of *p-*cresol (a phenol with structural and metabolic similarities to APAP) (Merrifield *et al.*, 2011; Thorn *et al.*, 2012). These results are consistent with the pig having been shown to have a reduced sulfation capacity compared with human (Capel *et al.*, 1972). Interestingly, the second most abundant metabolite measured in plasma and urine was PAP-G, which we propose to have been formed by the glucuronidation of PAP (Figure 5) following the N-deacetylation of APAP. That PAP-G was formed to a greater extent in the pig than in other species was presumably due to differences in the key drug metabolizing enzymes responsible for both the de and reacetylation of aromatic amides and amines such as APAP and PAP in this species. The production of PAP provides a ready explanation for the methemoglobin and nephrotoxicity observed in pigs dosed with APAP. Previously it has been suggested that the production of methemoglobin in pigs administered APAP was the result of erythrocyte GSH depletion and poor glucose uptake (Henne-Bruns *et al.*, 1988; Newsome *et al.*, 2010). However, the well-known association of aromatic amines, including PAP, with methemoglobin formation (Harrison and Jollow, 1987) provides a more obvious mechanism. Indeed, PAP has previously been suggested as the cause of APAP-induced methemoglobinemia cats and dogs (McConkey *et al.*, 2009). In most species, N-acetylation reactions in the liver are catalyzed by N-acetyl transferase (NAT) enzymes NAT1 and NAT2, whereas deacetylation is catalyzed by carboxylesterases (CES) including CES1 and CES2 and arylacetamide deacetylase (AADAC) in human. The rates of these de and reacetylation reactions have been shown to vary significantly amongst species (Jones *et al.*, 2016; McConkey *et al.*, 2009; Watanabe *et al.*, 2010). APAP has been shown to form PAP by N-deacetylation in man as a minor route of metabolism, and to a greater extent in rat with, in both cases, the majority being detoxified by rapid reacetylation to APAP in a so called “futile-deacetylation” reaction (Nicholls *et al.*, 1995). The formation of methemoglobin in the dog is attributed to a lack of either NAT1 or NAT2 enzymes and in cats to be the result of their only expressing NAT1 (McConkey *et al.*, 2009). C57BL6 mice have been shown to be slow to deacetylate APAP, but rapid to reacetylate and, whilst methemoglobin was formed as a result of APAP administration to NAT1/2 knockout mice, it was not comparable with the levels seen in dogs or cats. In addition to methemoglobinemia APAP administration to pigs in this study was associated with nephrotoxicity and the production of PAP represents a possible cause of this. For example, in rats, PAP has been reported to be a potent nephrotoxin (Gartland *et al.* 1990; Green *et al.*, 1969), primarily effecting the proximal tubules (Fowler *et al.*, 1993, 1994; Yang *et al.*, 2007), causing both tubular dilation and necrosis. Similar pathological changes were observed in this study for the pig. The exact mechanism of PAP-induced nephrotoxicity is not fully understood, however it has been postulated that the mechanism involves formation of PAP-glutathionyl conjugate as PAP-GSH has been reported to be more nephrotoxic than PAP itself (Fowler *et al.* 1991, 1993). Furthermore, PAP-GSH-induced nephrotoxicity has been reported to lead to glycosuria, which could explain the observed increase to glucose concentrations in the urine seen in the current work (Fowler *et al.*, 1991). γ-Glutamyltransferase (GGT) is predominantly localized in the brush border membrane of renal proximal tubules, the area susceptible to PAP and PAP-GSH-mediated nephrotoxicity. The inhibition of GGT has been reported to be protective against PAP-GSH-mediated nephrotoxicity (Fowler *et al.*, 1994). Studies utilizing suitably labeled APAP in the acetyl moiety as used previously (Nicholls *et al.* 1995, 1997) would be required to confirm the extent, if any, of futile deacetylation in the porcine model.

Despite clear ALF in these animals the relatively low amounts of GSH-derived APAP metabolites detected suggest, that this is not a major route in the pig. This is based, in part, on similar studies on mouse liver after APAP administration, where concentrations of APAP-GSH in aqueous extracts were comparable with those of APAG (Kyriakides *et al.*, 2016). However, the low concentrations of APAP-GSH detected in pig liver extracts, plasma and urine may conceivably result from other factors such as eg, rapid and extensive biliary excretion. However, it is clear from recent *in vitro* studies, in eg, HepG2 cells which are deficient in CYP450 activity, that APAP can also act as CYP metabolism-independent cytotoxin (Behrends *et al.*, 2019).This type of toxicity was linked to “a decoupling of glycolysis from the TCA cycle, lactic acidosis, reduced NADPH production and subsequent suppression of the anabolic pathways required for rapid growth” (Behrends *et al.*, 2019), providing a mechanism for cell death that is independent of oxidative stress. The clear hepatotoxicity observed here and in previous studies in the pig following APAP administration may therefore be the result of a combination of the effects of the production of reactive metabolites via NAPQI and direct cytotoxicity as seen in HepG2 cells. With respect to the study of human-relevant hepatotoxicity of APAP the mouse is perhaps the most appropriate model (Jaeschke *et al.*, 2014). However, the species differences in drug metabolism highlighted here for the pig go beyond those of the biotransformation of APAP and may significantly affect the toxicity profile of other drugs and xenobiotics with similar structural features.

## CONCLUSIONS

The pig is susceptible to hepatotoxicity, renal toxicity and metHb formation in response to paracetamol administration. The paracetamol-induced hepatotoxicity in the pig shows some histopathological similarity to that seen in humans, with a number of clinical similarities to APAP-induced ALF including signs of hepatic encephalopathy. In addition to hepatic toxicity, some renal toxicity was identified which may be related to the formation of PAP, as evidenced by relatively high concentrations of PAP-G in the liver, kidney, plasma, and urine of these animals. The pig showed low sulfation of APAP, producing mainly the phenolic glucuronide. In addition deacetylation to PAP, which was combined with formation of PAP-glucuronide and methemoglobinemia was observed. Only small amounts of the NAPQI-derived glutathione-related metabolites were detected. The metabolic differences observed here may mean that the pig represents a poor model for translational toxicity studies for humans for compounds undergoing high levels of sulfation. Similar concerns may also apply to studies with compounds that contain amide bonds which, if hydrolyzed, would lead to toxicity. The pig may however, be useful model where amide hydrolysis is seen for drugs or environmental chemicals in humans, but not in eg, rat and dog.

## SUPPLEMENTARY DATA

Supplementary data are available at *Toxicological Sciences* online.

## DECLARATION OF CONFLICTING INTERESTS

The authors declared no potential conflicts of interest with respect to the research, authorship, and/or publication of this article.

## Supplementary Material

kfaa023_Supplementary_Data
