# Supplementary material for: Metabolism and Effects on Endogenous Metabolism of Paracetamol (Acetaminophen) in a Porcine Model of Liver Failure
Source: Toxicol Sci. 2020 Apr 6;175(1):87–97. doi: 10.1093/toxsci/kfaa023 (PMC7197950; doi:10.1093/toxsci/kfaa023)
Supplement: kfaa023_Supplementary_Data [file kfaa023_supplementary_data.docx]

**Supplementary Material**

**Metabolism and Effects on Endogenous Metabolism of Paracetamol (Acetaminophen) in a Porcine Model of Liver Failure**

Rebecca Dargue^a^, Rabiya Zia^a^, Chungho Lau^a^, Andrew W. Nicholls^b^, Theo O. Dare^b^, Karla Lee^d^, Rajiv Jalan^e^, Muireann Coen^a,c*^, Ian D. Wilson^a^*

^a^ Division of Systems Medicine, Department of Metabolism, Digestion and Reproduction, Imperial College London, Exhibition Road, South Kensington, London SW7 2AZ, UK

^b^ GSK R&D, Park Road, Ware, Hertfordshire, SG12 0DP, UK

^c^ Oncology Safety, Clinical Pharmacology and Safety Sciences, IMED Biotech Unit, AstraZeneca, Unit 310, Cambridge Science Park, Milton Road, Cambridge, CB4 0WG, UK.

^d^ Department of Clinical Science and Services, Royal Veterinary College, University of London, Hertfordshire AL9 7TA , UK.

^e^ UCL Institute for Liver and Digestive Health, Royal Free Hospital, London NW3 2PF, UK.

*Joint senior authors

**Contents**

**Supplementary information 1:** Description of the Viamin 14 Glucose and AA solution. **Supplementary information 2:** Composition of Buffer solutions for NMR Spectroscopy. **Supplementary information 3:** Preparation of Standard Curve and QC stock solutions for UPLC-MS Methods. **Table S1.** Results for clinical chemistry tests on serum from pigs taken at 4 hr intervals up to ALF, and then at 4 hr intervals from ALF until death. **Table S2.** ^1^H NMR-detected Endogenous and APAP Metabolites in Plasma. **Table S3**. ^1^H NMR-detected Endogenous and APAP Metabolites in Urine. **Table S4.** ^1^H NMR-detected Endogenous and APAP Metabolites in Aqueous Liver Extracts. **Table S5.** ^1^H NMR-detected Endogenous and APAP Metabolites in Aqueous Kidney Extracts. **Table S6.** Metabolites Measured by UPLC-MS plus internal standards (where available) **Table S7.** Concentration ranges for APAP and metabolites in porcine plasma measured by UPLC-MS **Table S8.** QC concentrations used for the UPLC-MS of APAP and Metabolites in porcine plasma

**Table S9.** Volume of stock solutions and MeOH for preparation of stock solution A and B for the UPLC-MS of APAP and Metabolites. **Table S10.** Serial dilutions to produce Standard Curve stock solutions for the UPLC-MS of APAP and Metabolites. **Table S11.** Serial dilutions to produce QC stock solutions for the UPLC-MS of APAP and Metabolites. **Table S12.** Internal standard stock solution preparation for UPLC-MS of APAP and Metabolites. **Table S13.** UPLC-MS/MS conditions for analytes and internal standards used for the UPLC-MS of APAP and Metabolites.

**Figure S1:** Overview of study design, timings and sampling. **Figure S2:** Histopathology images **Figure S3:** Representative ^1^H NMR spectra of porcine plasma at the ALF time point. **Figure S4:** Representative ^1^HNMR spectra of porcine urine at the ALF time point. **Figure S5**: Significant changes to endogenous metabolites measured in plasma (^1^H NMR). **Figure S6**: Significant changes to endogenous metabolites measured in urine (^1^H NMR). **Figure S7**. Significant changes to endogenous metabolites measured in liver extracts (^1^H NMR). **Figure S8.** Significant changes to endogenous metabolites measured in kidney extracts (^1^H NMR). **Figure S9.** APAP and metabolites measured by ^1^H-NMR spectroscopy in biofluids and tissue extracts. **Figure S10.** Sample, Standard Curve and QC Stock Solution Preparation used for the UPLC-MS of APAP and Metabolites in porcine plasma. **Figure S11**. Sequence of UPLC-MS analysis for APAP/metabolite quantification of randomized samples bracketed by calibration standards and interspersed with QC injections. **Figure S12.** Chromatogram showing overlaid peaks for each of the metabolites measured by the method. **Figure S13**. Representative total ion chromatogram of APAP metabolites in porcine plasma after 4 hours of dosing.

**Supplementary information 1: Viamin 14**

The Viamin 14 Glucose and AA solution contained alanine, arginine, aspartic acid, cysteine, glutamic acid, glycine, histidine, isoleucine, leucine, lysine, methionine, phenylalanine, proline, serine, threonine, tryptophan, tyrosine, valine, glacial acetic acid.

**Supplementary information 2: Buffer solutions for NMR Spectroscopy**

**Urine buffer** (1.5M KH_2_PO_4_) was prepared by dissolving 20.4g of KH_2_PO_4_ in 80mL of D_2_O. TSP (100mg) and NaN_3_ (13mg) was dissolved in 10mL of D_2_O. Both solutions were mixed thoroughly, and then mixed together. The buffer pH was adjusted to 7.4 by adding KOH pellets, volume was adjusted to 100mL by adding D_2_O and the pH was rechecked.

**Tissue buffer** was prepared by mixing TSP (0.009g), NaH_2_PO_4_ (1.2g) and NaN_3_ (0.2g) with 80mL of D_2_O. The solution was mixed thoroughly, and the pH was adjusted to 7.4 by adding KOH pellets, volume was adjusted to 100mL by adding D_2_O and the pH was rechecked.

**Plasma buffer**: 100 mM sodium phosphate buffer in 100% D_2_O, pH 7.4, containing 0.5 mM TSP and 0.2% w/v NaN_3_

**Supplementary Information 3: Preparation of Standard Curve and QC stock solutions for UPLC-MS Methods.**

The method was developed using the standards as listed in **Table S6**. For all metabolites except APAP-GSH and PAP-G (due to lack of availability) a deuterium labelled internal standard was used to account for ion suppression and to aid with quantification. The method was validated to FDA standards with a cross validation to porcine plasma (Dargue *et al* in preparation) over the standard curve and QC ranges listed in **Tables S7-8**. Standards in solution 50:50 H_2_O: MeOH (v/v) at the concentrations in parentheses (**Table S9**) were mixed in methanol to form a top ‘stock A’ for the curve and for the QCs, which was diluted to form ‘stock B’ or the ULOQ (**Table S11**). This Stock B was serially diluted with MeOH as per tables **S12** and **S13** for the standard curves and QCs respectively.


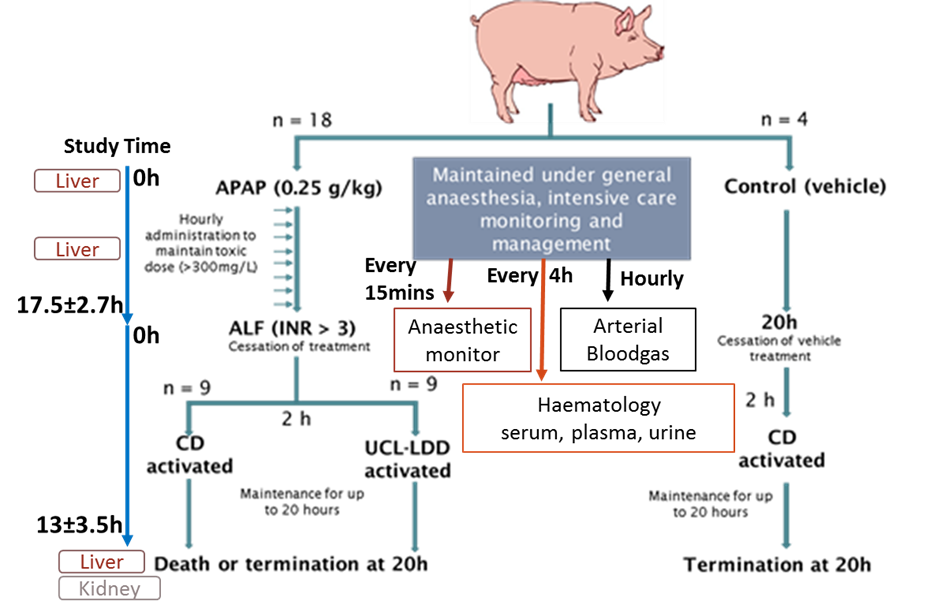


**Figure S1**. Overview of study design, sample collection time-points . UCL-LDD=UCL Liver Dialysis Device, CD= Control Device, INR=International Normalised Ratio.

As described in Lee et al. (2015) (Extracorporeal liver assist device to exchange albumin and remove endotoxin in acute liver failure: Results of a pivotal pre-clinical study. J. Hepatol. 63:634-642 doi: 10. 1016 /j.jhep. 2015.04.020) all four Control-CD, 9 APAP-UCL-LDD and 7 (out of 9) APAP-CD pigs completed the study. However, 2 Of the pigs fitted with the APAP-CD device 2 reached ALF criteria at 12.5 and 13 h after initiation of APAP dosing (30% below the mean of 19.3 ± 1.8 h expected for this model) and were excluded. However, for the remaining APAP-UCL-LDD and APAP-CD animals the mean time to ALF = 18.4 ± 0.6 h (mean cumulative doses = 50.7 ± 3.0 g for the APAP-CD and 49.2 ± 4.0 g for the APAP-UCL-LDD animals respectively) and these animals were allowed to complete the study to compare the UCL-LDD and CD interventions as described in Lee et al 2015.

| time | Urea  mmol/L | | Creatinine  µmol/L | | Total Bilirubin µmol/L | | | AST  U/L | | ALP  U/L | | Albumin  g/L | |
| --- | --- | --- | --- | --- | --- | --- | --- | --- | --- | --- | --- | --- | --- |
|  | A | C | A | C | A | | C | A | C | A | C | A | C |
| 0h | 4±1 | 4±1 | 65±9 | 56±9 | 2±1 | 3±2 | | 39±13 | 43±13 | 203±36 | 184±70 | 31±3 | 34±2 |
| 4h | 4±1 | 4±0.4 | 67±7 | 58±11 | 4±1 | 5±2 | | 49±16 | 47±12 | 160±27 | 160±56 | 24±3 | 30±3 |
| 8h | 4±1 | 3±1 | 73±8* | 53±9 | 6±1 | 6±2 | | 54±15 | 53±28 | 154±28 | 129±36 | 22±4 | 29±3 |
| 12h | 4±1 | 3±0.5 | 75±9 | 61±11 | 6±2 | 4±2 | | 51±17 | 61±12 | 132±28 | 133±51 | 18±4 | 29±1* |
| 16h | 4±1 | 3±0.3 | 95±24* | 65±12 | 9±1 | 6±1 | | 34±11 | 45±21 | 85±28 | 135±45 | 21±2 | 29±6* |
| ALF | 4±1 | 3±0.3 | 114±24* | 66±9 | 5±1 | 6±2 | | 31±11 | 62±13 | 62±28 | 133±42 | 13±2 | 30±6* |
| ALF+4h | 4±1 | 3±0.4 | 107±20* | 61±16 | 5±1 | 9±4 | | 44±12 | 97±19 | 51±14 | 107±27 | 16±2 | 30±6* |
| ALF+8h | 3±1 | 3±0.8 | 103±18* | 49±22 | 6±1 | 7±6 | | 45±16 | 79±28 | 51±19 | 91±23 | 17±3 | 27±7* |
| ALF+12h | 3±1 | 2±0.6 | 104±20* | 45±15 | 6±2 | 6±3 | | 52±19 | 86±19 | 68±46 | 98±25 | 19±5 | 29±4* |
| ALF+16h | 3±1 | 2±0.8 | 102±13* | 35±16 | 9±2 | 6±2 | | 88±35 | 71±10 | 77±22 | 85±23 | 24±4 | 28±3 |
| ALF+20h | 3±1 | 2±0.6 | 101±9* | 41±10 | 10±2 | 6±1 | | 124±27 | 70±9 | 100±39 | 88±23 | 26±5 | 31±2 |

**Clinical Chemistry Results**

**Table S1.** **Results for clinical chemistry tests on serum from pigs taken at 4 hr intervals up to ALF, and then at 4 hr intervals from ALF until death.** Mean + SD; Asterisk (*) indicates where A=APAP treated (n=18) C=control (n=4), APAP-treated animals were significantly different to controls with a q value <0.05 after FDR correction, following Kruskal-Wallis with multiple comparisons

Adapted from Lee KCL, Baker LA, Stanzani G, et al. (2015) Extracorporeal liver assist device to exchange albumin and remove endotoxin in acute liver failure: Results of a pivotal pre-clinical study. J. Hepatol. 63:634-642 doi:10.1016/j.jhep.2015.04.020

**Liver histopathology**

Histopathology of post mortem liver specimens showed acute centrilobular to midzonal hepatocyte degeneration and necrosis in all APAP-UCL-LDD and APAP-CD pigs. These changes were not seen in Control-CD pigs. Based on the degree and percentage of parenchyma affected, necrosis was graded either as mild, moderate or severe. For both APAP-UCL-LDD and APAP-CD animals the median grade was moderate, with 4/9 APAP-UCL-LDD pigs and 3/7 APAP-CD showing severe necrosis. Representative examples of histopathology are shown in **Figure S2**.

| **A) i** **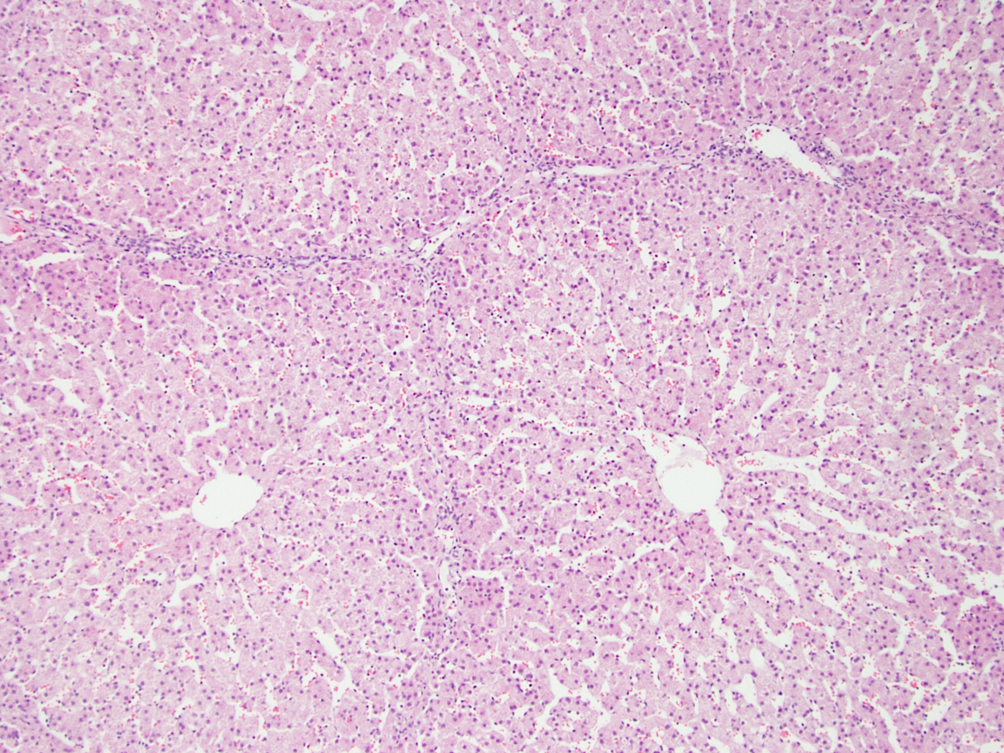** | **A) ii** **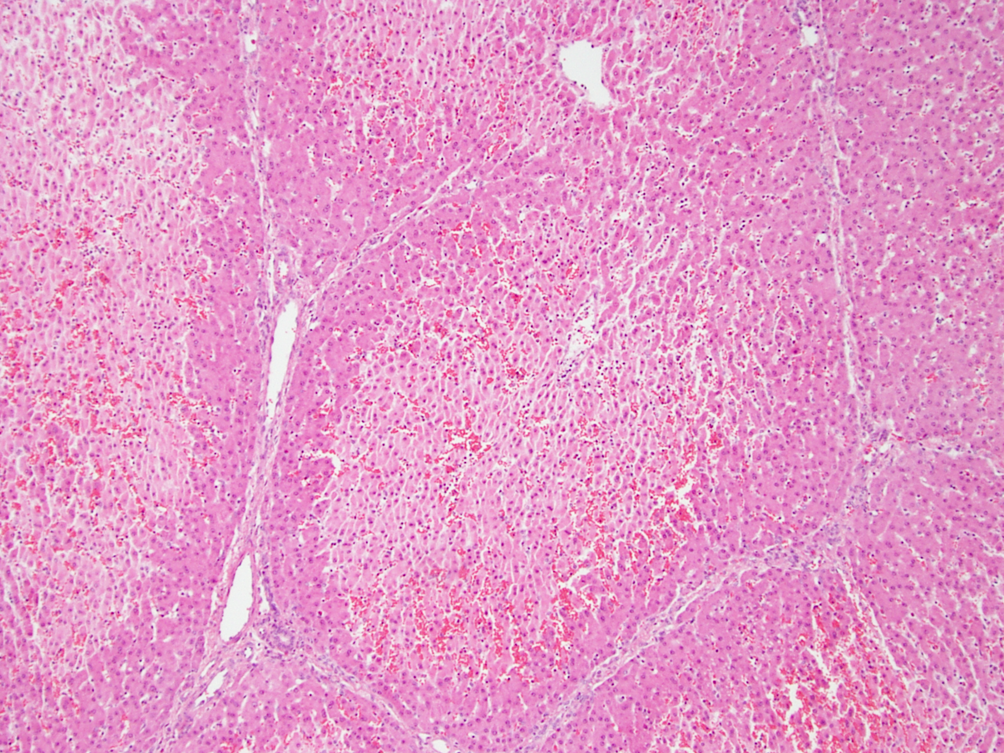** |
| --- | --- |
| **B) i** **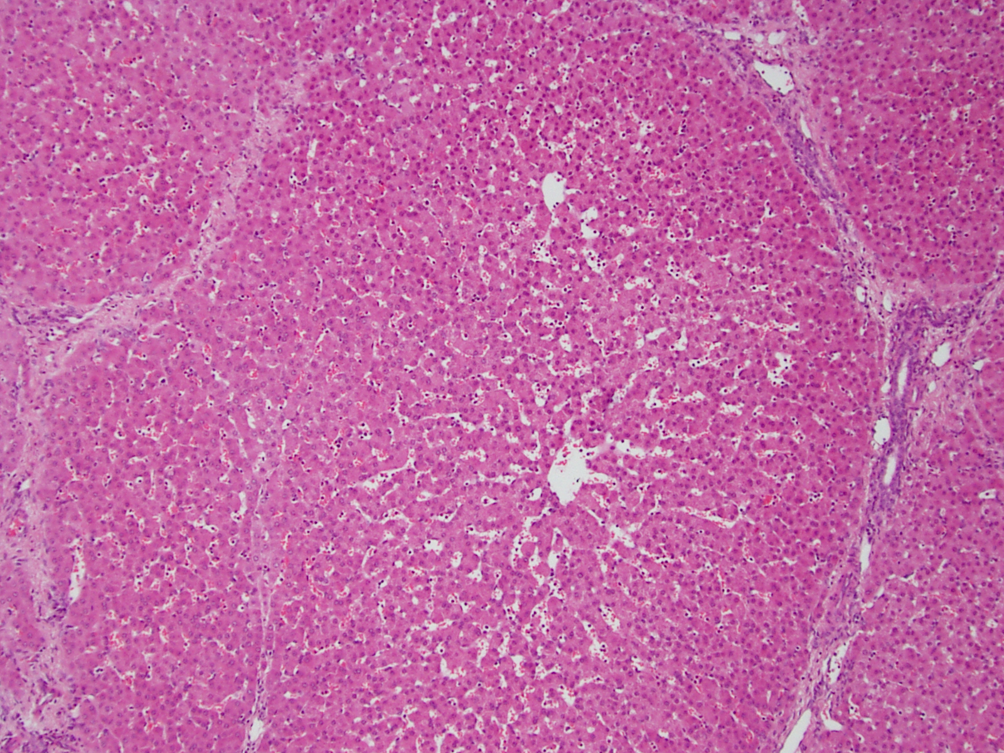** | **B) ii**  **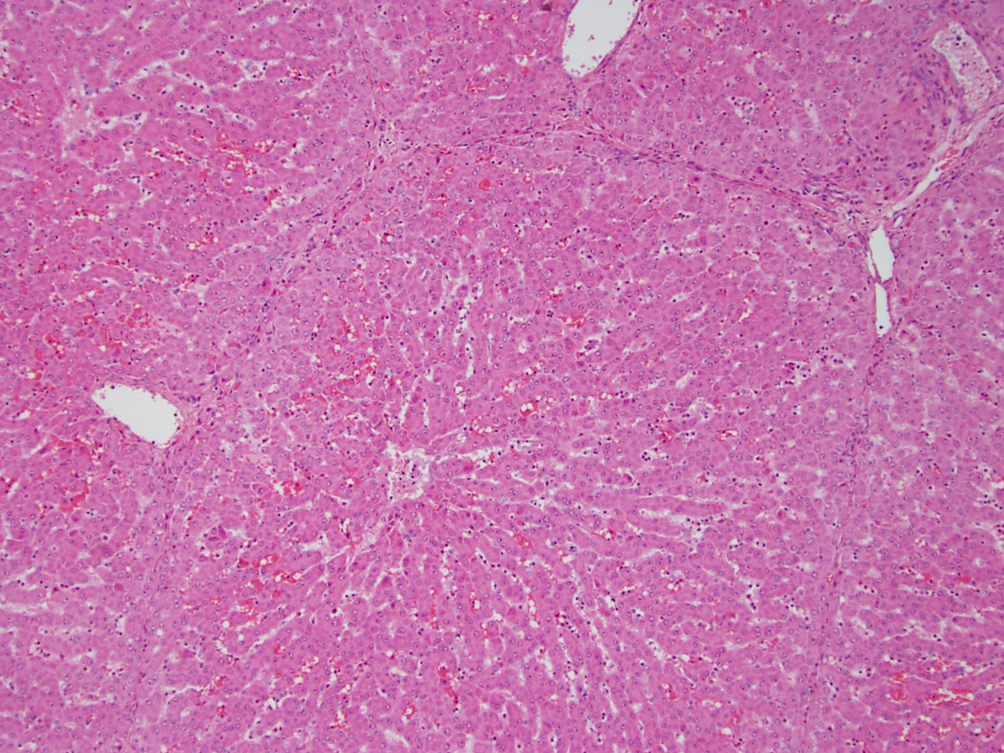** |
| **C) i** **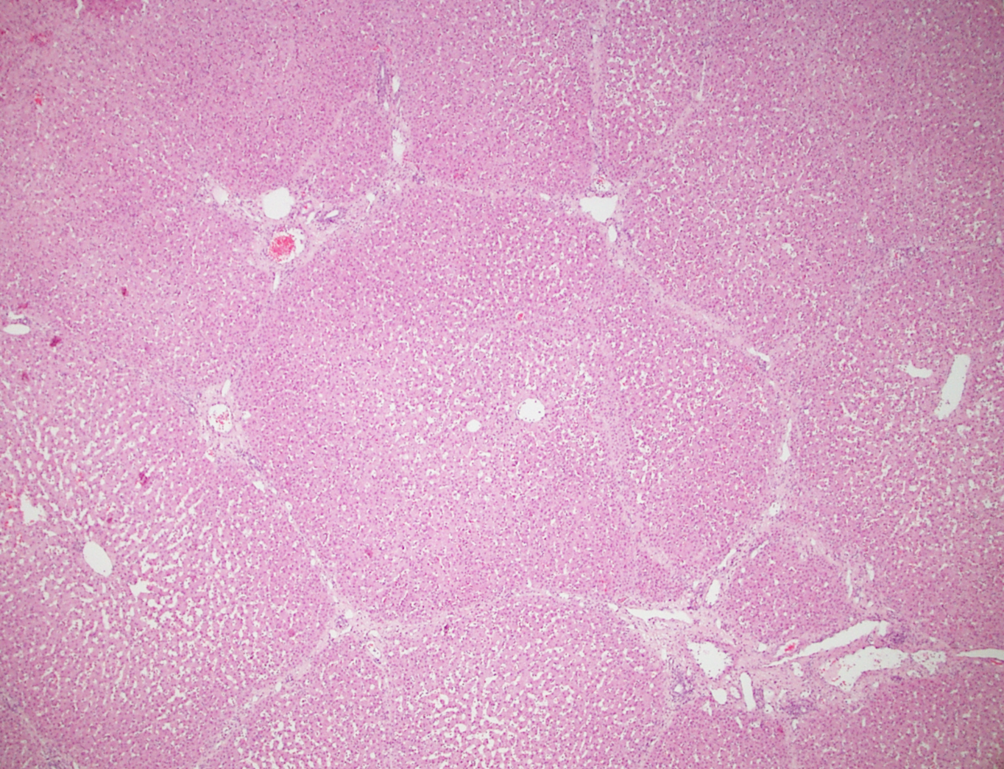** | **C) ii** **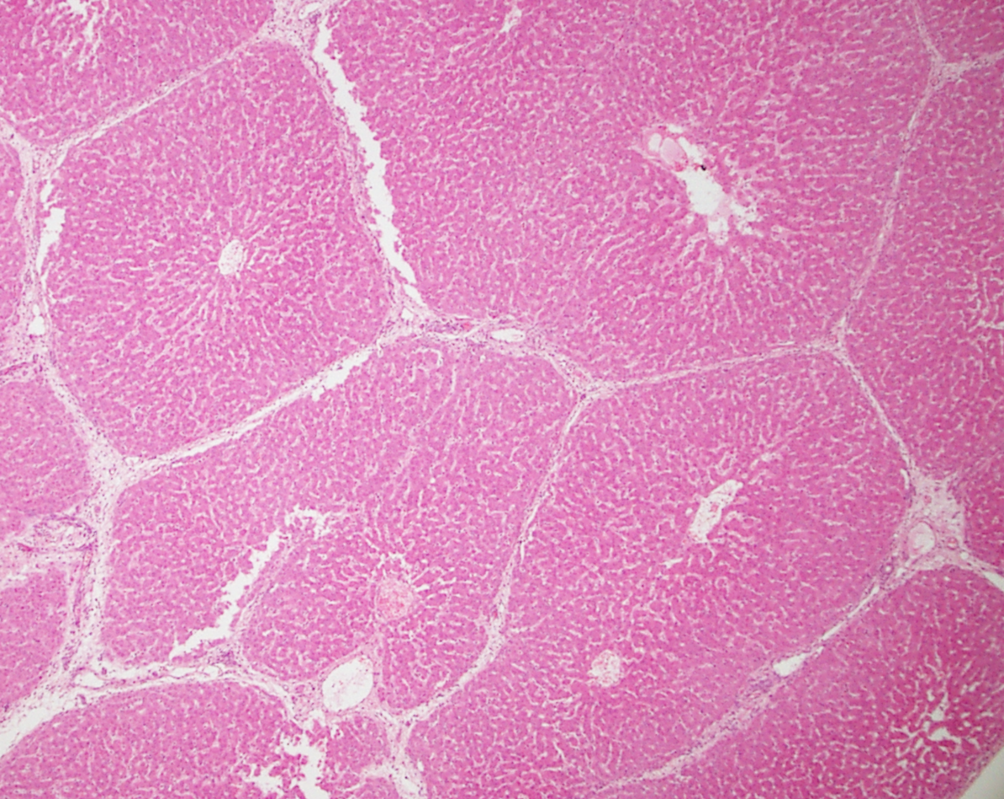** |

**Figure S2** Histopathology of pigs before APAP dosing (i) and at post-mortem (ii). A) a Control-CD pig demonstrating normal liver at post-mortem. B) An APAP-UCL-LDD pig demonstrating multifocal, moderate centrilobular to midzonal hepatocellular degeneration and rare necrosis with mild sinusoidal congestion at post-mortem. C) an APAP-CD pig demonstrating severe centrilobular to midzonal hepatocellular degeneration and necrosis with sinusoidal haemorrhage and congestion at post-mortem (x40 magnification, haematoxylin and eosin stain). Adapted from supplementary data provided for Lee KCL, Baker LA, Stanzani G, et al. (2015) Extracorporeal liver assist device to exchange albumin and remove endotoxin in acute liver failure: Results of a pivotal pre-clinical study. J. Hepatol. 63:634-642 doi:10.1016/j.jhep.2015.04.020

**^1^H NMR Data**

**Table S2.** ^1^H NMR-detected Endogenous and APAP Metabolites in Plasma

| **Plasma** | | | |
| --- | --- | --- | --- |
| **Endogenous metabolites** | | | |
| **Metabolite** | **ppm region** | **Multiplicity** | **Functional group** |
| Acetate | 1.92-1.93 | s | βCH_3_ |
| alanine | 1.47-1.50 | d | βCH_3_ |
| Citrate | 2.50 -2.57 | d | 1/2γCH |
| Creatine/creatinine | 3.04-3.06 | S/S | N-CH_3_ |
| Formate | 8.45-8.48 | s | HCOOH |
| α-glucose | 5.22-5.28 | d | C1H |
| glutamine | 2.44-2.48 | m | γCH_3_ |
| isobutyrate | 1.13-1.14 | d | CH_3_ |
| lactate | 4.10-4.15 | q | αCH |
| Phenylalanine | 7.42-7.47 | m | C3H/C6H |
| Proline | 4.17-4.21 | dd | αCH |
| Pyruvate | 2.38-2.39 | s | CH_3_ |
| Tyrosine | 7.19-7.22 | d | C2H/C6H |
| Valine | 1.03-1.06 | d | γCH_3_ |
| **APAP metabolites** | | | |
| APAP | 7.24 -7.29 | d | C2H/C6H |
| APAP-G | 7.14-7.18 | d | C3H/C5H |
| PAP-G | 6.82-6.86 | d | C3H/C5H |


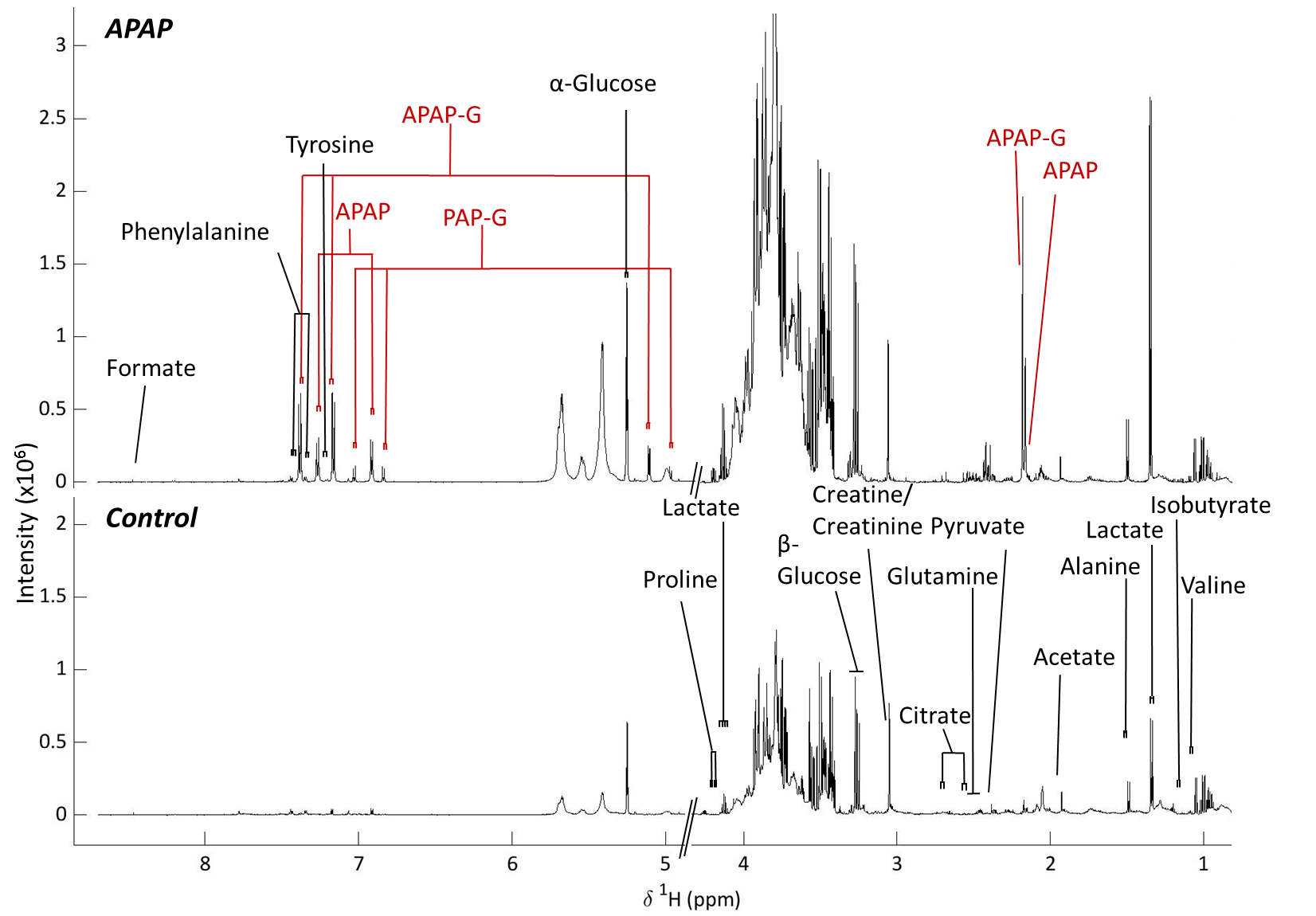
 **Figure S3** Representative ^1^H NMR spectra of porcine plasma at the ALF time point in an APAP treated and Control animal at post mortem with key endogenous and drug metabolite resonances labelled.

**Table S3**. **^1^H NMR-detected** **Endogenous and APAP Metabolites in Urine**

| **Urine** | | | |
| --- | --- | --- | --- |
| **Endogenous metabolites** | | | |
| **Metabolite** | **ppm region** | **Multiplicity** | **Functional group** |
| alanine | 1.47-1.50 | d | βCH_3_ |
| choline | 3.26-3.27 | s | N-(CH_3_)_3_ |
| creatine | 2.00-3.02 | s | N-CH_3_ |
| creatinine | 3.02-3.04 | s | N-CH_3_ |
| dimethylamine | 2.71-2.72 | s | CH_3_ |
| α-glucose | 5.23-5.27 | d | C1H |
| β-glucose | 4.64-4.69 | d | C1H |
| lactate | 1.31-1.36 | d | βCH_3_ |
| phosphocholine | 3.27-3.27 | s | N-(CH_3_)_3_ |
| glycero-phosphocholine | 3.27-3.28 | s | N-(CH_3_)_3_ |
| trimethylamine | 2.87-2.90 | s | CH_3_ |
| TMAO | 3.28-3.30 | s | N-(CH_3_)_3_ |
| **APAP metabolites** | | | |
| **Metabolite** | **ppm region** | **Multiplicity** |  |
| APAP | 6.86-6.92 | d | C3H/C5H |
| APAP-Cys | 6.93-6.98 | d | C5H |
| APAP-G | 7.09-7.15 | d | C3H/C5H |
| APAP-S | 7.29-7.32 | d | C2H/C6H |
| PAP-G | 6.80-6.85 | d | C3H/C5H |


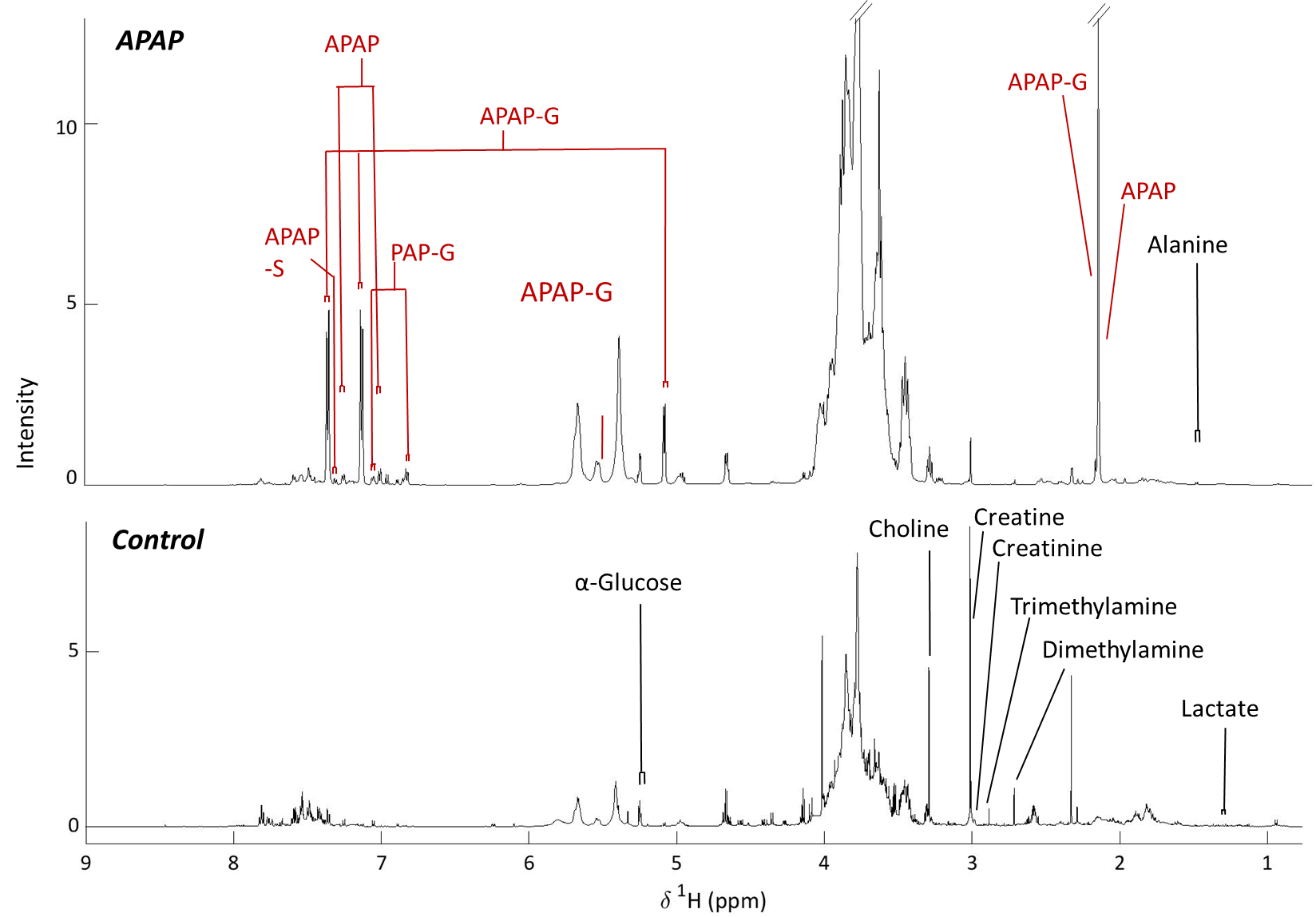


**Figure S4**. Representative ^1^HNMR spectra of porcine urine at the ALF time point in an APAP treated and Control animal with key endogenous and drug metabolite resonances labelled.

**Table S4. ^1^H NMR-detected Endogenous and APAP Metabolites in Aqueous Liver Extracts**

| **Liver** | | | |
| --- | --- | --- | --- |
| **Endogenous metabolites** | | | |
| **Metabolite** | **ppm region** | **Multiplicity** | **Functional group** |
| acetate | 1.91-1.94 | s | βCH_3_ |
| alanine | 1.47-1.50 | d | βCH_3_ |
| AMP | 8.27-8.29 | s | C8H |
| choline | 3.20-3.21 | s | N-(CH_3_)_3_ |
| creatine | 3.03-3.05 | s | N-CH_3_ |
| formate | 8.45-8.48 | s | HCOOH |
| α-glucose | 5.22-5.26 | d | C1H |
| β-glucose | 4.63-4.67 | d | C1H |
| inosine | 8.33-8.36 | s | C8H |
| isobutyrate | 1.13-1.16 | d | CH_3_ |
| lactate | 1.31-1.35 | d | βCH_3_ |
| phosphocholine | 3.22-3.23 | s | N-(CH_3_)_3_ |
| glycero-phosphocholine | 3.23-3.24 | s | N-(CH_3_)_3_ |
| succinate | 2.40-2.41 | s | CH_2_ |
| TMAO | 3.26-3.27 | s | N-(CH_3_)_3_ |
| **APAP metabolites** | | | |
| APAP | 7.25-7.27 | d | C2H/C6H |
| APAP-G | 7.14-7.16 | d | C3H/C5H |
| APAP-GSH | 6.95-6.97 | d | C5H |
| PAP-G | 6.79-6.80 | d | C3H/C5H |

**Table S5. ^1^H NMR-detected Endogenous and APAP Metabolites in Aqueous Kidney Extracts**

| **Kidney** | | | |
| --- | --- | --- | --- |
| **Endogenous metabolites** | | | |
| **Metabolite** | **ppm region** | **Multiplicity** | **Functional group** |
| acetate | 1.90 - 1.94 | s | βCH_3_ |
| alanine | 1.47 - 1.50 | d | βCH_3_ |
| choline | 3.19 - 3.21 | s | N-(CH_3_)_3_ |
| creatine | 3.03 - 3.05 | s | N-CH_3_ |
| α-glucose | 5.22 - 5.26 | D | C1H |
| β-glucose | 4.63 - 4.69 | D | C1H |
| lactate | 1.26 - 1.39 | d | βCH_3_ |
| phosphocholine | 3.22 - 3.23 | s | N-(CH_3_)_3_ |
| succinate | 2.40 - 2.42 | s | CH_2_ |
| TMAO | 3.26 - 3.28 | s | N-(CH_3_)_3_ |
| **APAP metabolites** | | | |
| APAP | 7.24 - 7.27 | d | C2H/C6H |
| APAP-Cys | 6.95 - 6.996 | d | C5H |
| APAP-G | 7.12 - 7.18 | d | C3H/C5H |
| PAP-G | 7.00 - 7.04 | D | C2H/C6H |

**Endogenous metabolite results**

**Plasma**

**Figure S5.** Significant changes to endogenous metabolites measured in plasma (^1^H NMR). Kruskall-Wallis with FDR corrected mulitple comparisons. * q <0.05.

**Urine**

**Figure S6.** Endogenous metabolite changes in urine (^1^H NMR). Kruskall-Wallis with FDR corrected mulitple comparisons. * q <0.05.

**Liver**

**Figure S7**. Endogenous metabolite changes in Liver extracts (^1^H NMR). Kruskall-Wallis with FDR corrected mulitple comparisons. * q <0.05.

**Kidney**

**Figure S8**. Significant changes to endogenous metabolites in kidney extracts (^1^H NMR). Mann-Whitney **p<0.01 ***p<0.001

A B

C D

**Figure S9.** APAP and metabolites measured by ^1^H-NMR spectroscopy in biofluids and tissue extracts. A) Plasma: median concentration and interquartile range of APAP and 2 metabolites identified in plasma over the time course of the experiment (n=18). B) Urine: Median concentration and interquartile range of APAP and 4 metabolites identified in samples across the time course of the experiment (n=12) C) Liver aqueous extracts: APAP plus 3 metabolites identified in samples taken at predose (n=17), 12 hours (n=3) and at post mortem (n=17) Boxes represent median and interquartile range while whiskers represent the full range, all data points are shown. D) Kidney aqueous extracts: APAP and 3 metabolites identified in samples taken post mortem (n=18). Boxes represent median and interquartile ranges while whiskers represent the full range, all data points are shown.

**UPLC-MS**

**Table S6. Metabolites Measured by UPLC-MS plus internal standards (where available)**

| APAP metabolites | Internal Standard |
| --- | --- |
| APAP | APAP-d_3_ |
| APAP-Sulfate | APAP-Sulfate-d_3_ |
| APAP-Glucuronide | APAP-Glucuronide-d_3_, |
| APAP-Cysteinyl | APAP-Cysteinyl-d_5_ |
| APAP-NAC | APAP-NAC-d_5_ |
| APAP-GSH | Not available |
| PAP-G | Not available |

**Table S7. Concentration ranges for APAP and metabolites in porcine plasma measured by UPLC-MS**

| Calibration Curve Ranges / ng/ml | | | | | |
| --- | --- | --- | --- | --- | --- |
| APAP, PAP-G | APAP-G | APAP-S | APAP-C | APAP-SG | APAP-NAC |
| 16 | 22.5 | 2.75 | 0.44 | 0.58 | - |
| 24 | 33.8 | 4.12 | 0.67 | 0.88 | 0.84 |
| 40 | 56.3 | 6.87 | 1.11 | 1.46 | 1.40 |
| 60 | 84 | 10.3 | 1.67 | 2.19 | 2.10 |
| 90 | 127 | 15.5 | 2.50 | 3.28 | 3.16 |
| 150 | 211 | 25.8 | 4.173 | 5.47 | 5.26 |
| 250 | 352 | 42.9 | 6.94 | 9.12 | 8.77 |
| 500 | 703 | 85.9 | 13.9 | 18.2 | 17.5 |

**Table S8. QC concentrations used for the UPLC-MS of APAP and Metabolites in porcine plasma**

| QC Concentration in ng/ml | | | | | | |
| --- | --- | --- | --- | --- | --- | --- |
| QC | APAP-G | APAP, PAP-G | APAP-S | APAP-SG | APAP-C | APAP-NAC |
| ULOQ | 703 | 500 | 85.9 | 18.2 | 13.9 | 17.5 |
| High | 562 | 400 | 68.7 | 14.6 | 11.1 | 14.0 |
| Mid | 141 | 100 | 17.2 | 3.65 | 2.78 | 3.51 |
| Low | 67.5 | 48 | 8.24 | 1.75 | 1.33 | 1.68 |
| LLOQ | 22.5 | 16 | 2.75 | 0.58 | 0.44 | 0.84 |

**Table S9. Volume of stock solutions and MeOH for preparation of stock solution A for the UPLC-MS of APAP and Metabolites.**

Concentrations of each stock solution prepared to create the standards for stock solution A and dilution to form stock solution B

| Standard added to Stock Soln. A from 1mg/mL standard stock solutions | Volume stock soln. added to make up to 1mL total in MeOH(µL) | Concentration of each individual standard in stock soln.A (µg/ml) | Final concentration of standards in ‘**Stock Soln.B**’ (ULOQ stock) following 1 in 5 dilution in MeOH (µg/ml) |
| --- | --- | --- | --- |
| APAP (1mg/mL) | 250 | 250 | 50.0 |
| APAP-S (2.5mg/mL) | 20 | 43 | 8.59 |
| APAP-G (1mg/mL) | 375 | 351 | 70.3 |
| APAP-C (1mg/mL) | 10 | 6.95 | 1.39 |
| APAP-NAC (1mg/mL) | 10 | 8.75 | 1.75 |
| APAP-SG (1mg/mL) | 10 | 9.1 | 1.82 |
| PAP-G (1mg/mL) | 250 | 250 | 50.0 |
| *MeOH added* | *75* | *240* |  |

**Table S10. Serial dilutions to produce Standard Curve stock solutions for UPLC-MS of APAP and Metabolites.**

| **Curve stock number** | **Stock soln. used** | **Volume of stock soln used (µL)** | **Volume of MeOH added (µL)** | **Final Concentration of individual curve stocks (**n**g/mL)** | | | | | | |
| --- | --- | --- | --- | --- | --- | --- | --- | --- | --- | --- |
|  |  |  |  | APAP-G, | APAP, PAP-G | APAP-S, | APAP-C | APAP-SG, | | APAP-NAC |
| **1 (Stock soln B)** | 1 (**Stock B)** | | | 70.3 | 50.0 | 8.59 | 1.390 | 1.820 | 1.750 | |
| **2** | 1 | 300 | 300 | 35.2 | 25.0 | 4.29 | 0.694 | 0.912 | 0.877 | |
| **3** | 2 | 300 | 200 | 21.2 | 15.0 | 2.58 | 0.417 | 0.547 | 0.526 | |
| **4** | 3 | 300 | 200 | 12.7 | 9.00 | 1.55 | 0.250 | 0.328 | 0.316 | |
| **5** | 4 | 300 | 200 | 8.4 | 6.00 | 1.03 | 0.167 | 0.219 | 0.210 | |
| **6** | 5 | 300 | 150 | 5.63 | 4.00 | 0.687 | 0.111 | 0.146 | 0.140 | |
| **7** | 6 | 300 | 200 | 3.38 | 2.40 | 0.412 | 0.067 | 0.088 | 0.084 | |
| **8** | 7 | 200 | 100 | 2.25 | 1.60 | 0.275 | 0.044 | 0.058 | - | |

**Table S11. Serial dilutions to produce QC stock solutions.**

| **QC** | **Std. used** | **Volume of Std used (µL)** | **Vol. of MeOH added (µL)** | QC Concentration in ng/ml | | | | | |
| --- | --- | --- | --- | --- | --- | --- | --- | --- | --- |
|  |  |  |  | APAP-G | APAP, PAP-G | APAP-S | APAP-SG | APAP-C | APAP-NAC |
| ULOQ (**Stock Solution B)** | | | | 703 | 500 | 85.9 | 18.2 | 13.9 | 17.5 |
| High | ULOQ | 800 | 200 | 562 | 400 | 68.7 | 14.6 | 11.1 | 14.0 |
| Mid | High | 250 | 750 | 141 | 100 | 17.2 | 3.65 | 2.78 | 3.51 |
| Low | Mid | 480 | 520 | 67.5 | 48 | 8.24 | 1.75 | 1.33 | 1.68 |
| LLOQ 2 | Low | 500 | 500 | 33.8 | 24 | 4.12 | 0.875 | 0.665 | 0.84 |
| LLOQ 1 | LLOQ 2 | 500 | 250 | 22.5 | 16 | 2.75 | 0.58 | 0.44 | - |

**Table S12. Internal standard stock solution preparation**

| Individual Stock Solution  Added to stock A | Volume stock soln. added to make up to 1mL total in MeOH(µL) | I.S. Stock Soln. A Concentration  (µg/mL) | Final I.S. Stock Soln. concentration (1 in 5 dilution) (µg/mL) | Final concentration measured by instrument (1 in 500 dilution) (ng/mL) |
| --- | --- | --- | --- | --- |
| APAP-d3 (1mg/mL) | 500 | 500 | 100 | 200 |
| APAP-S-d3 (1mg/mL) | 100 | 100 | 20 | 40 |
| APAP-G-d3 (2.5mg/mL) | 300 | 750 | 150 | 300 |
| APAP-C-d5 (1mg/mL) | 30 | 30 | 6 | 12 |
| APAP-NAC-d5 (1mg/mL) | 30 | 30 | 6 | 12 |
| *MeOH* | *40* | - | - | - |
| ***Total*** | ***1000*** | ***-*** | ***-*** | ***-*** |

Individual internal standard solutions were prepared by diluting 1mg standard into 1mL 50:50 or MeOH:H2O with the exception of APAP-G which was prepared at 2.5mg/mL. Standards were added together to make 1mL of mixed internal standard stock solution A, which was then diluted further by adding 500µL stock solution to 2000µL MeOH to produce the final internal standard stock solution.

**Table S13. UPLC-MS/MS conditions for analytes and internal standards**

| Compound | Parent ion (m/z) | Product ions  (m/z) | Cone voltage (V) | Collision voltage (V) | Dwell time (s) | Retention Time (min) |
| --- | --- | --- | --- | --- | --- | --- |
| APAP | 152.1000 | 65.0000  92.8800  110.0600 | 30 | 26  22  16 | 0.02 | 2.92 |
| APAP-D3 | 155.0700 | 110.9960 | 30 | 20 | 0.089 | 2.90 |
| APAP-cysteine | 271.0319 | 96.0944  139.9905  182.0616 | 34 | 36  24  16 | 0.02 | 2.52 |
| APAP-cysteine D5 | 276.2235 | 142.8145  186.8418 | 34 | 26  12 | 0.02 | 2.49 |
| APAP-sulphate | 232.07 | 110.0900  152.1000 | 30 | 22  12 | 0.02 | 2.18 |
| APAP-sulphate D3 | 235.0166 | 111.0218  155.0525 | 30 | 22  14 | 0.02 | 2.16 |
| APAP-glucuronide | 328.13 | 110.0080  152.0900 | 20 | 34  14 | 0.02 | 1.80 |
| APAP-glucuronide D5 | 353.2042 | 353.2042  369.1404 | 42 | 16  26 | 0.02 | 1.78 |
| APAP-NAC | 335.1404 | 152.0276  206.0525 | 6 | 16  18 | 0.08 | 4.98 |
| APAP-NACD5 | 340.2042 | 152.0235  211.0588 | 34 | 18  16 | 0.08 | 4.95 |
| APAP-GSH | 457.2466 | 139.9978  328.1093 | 30 | 36  14 | 0.089 | 3.85 |
| PAP-G | 308.33 | 181.04  199.05 | 2 | 12  12 | 0.025 | 0.39 |


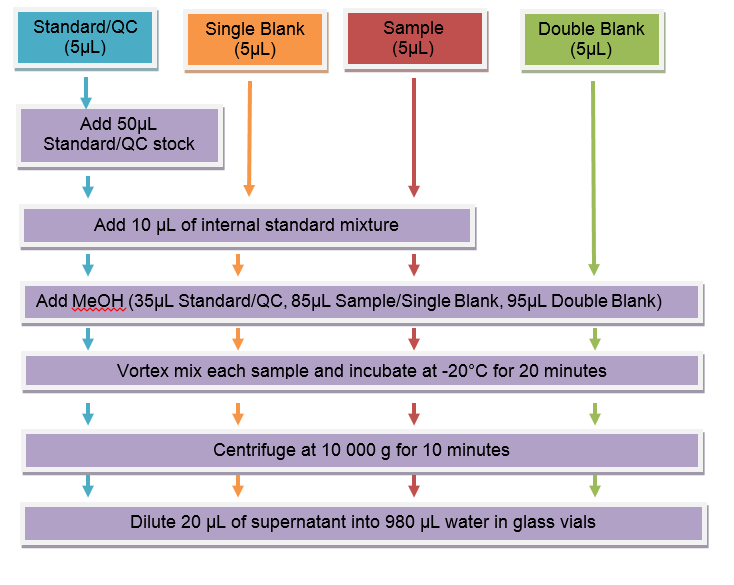


**Figure S10.** Sample, standard curve and QC Stock Solution preparation used for the UPLC-MS of APAP and metabolites in porcine plasma

**Sample Analysis**

For analysis, 2 µL of sample were injected first in order to confirm system cleanliness. Then 2 µL of the single blank was injected, followed by 2 µL injections of the calibration curve samples (low concentrations to high) followed by a double blank sample and then the study samples (randomized prior to sample preparation to minimize bias during analysis). The LQC, MQC and HQC (at least 6 QC samples, 2 at each concentration) interspersed evenly throughout the study samples, were analyzed. Following analysis of the study samples and QCs a second set of calibration samples were also analyzed. The analytical run sequence is summarized in **Figure S2**.


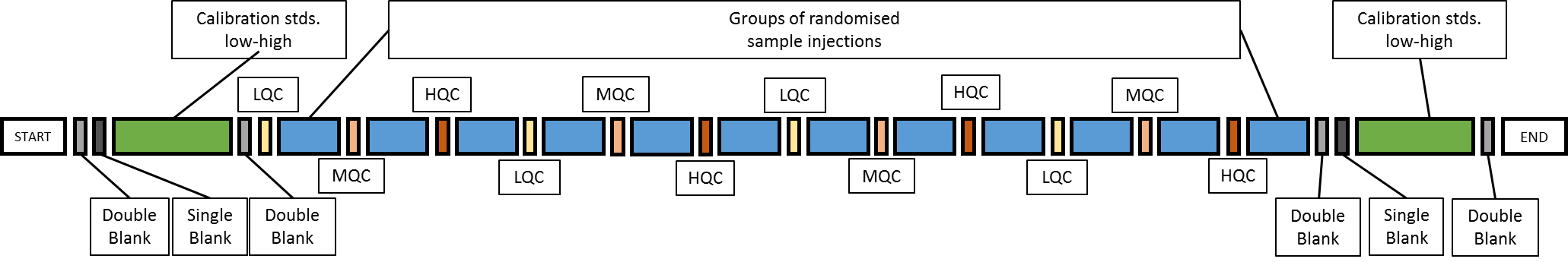


**Figure S11**. Sequence of UPLC-MS analysis for APAP/metabolite quantification of randomized samples bracketed by calibration standards and interspersed with QC injections.

**Chromatography**


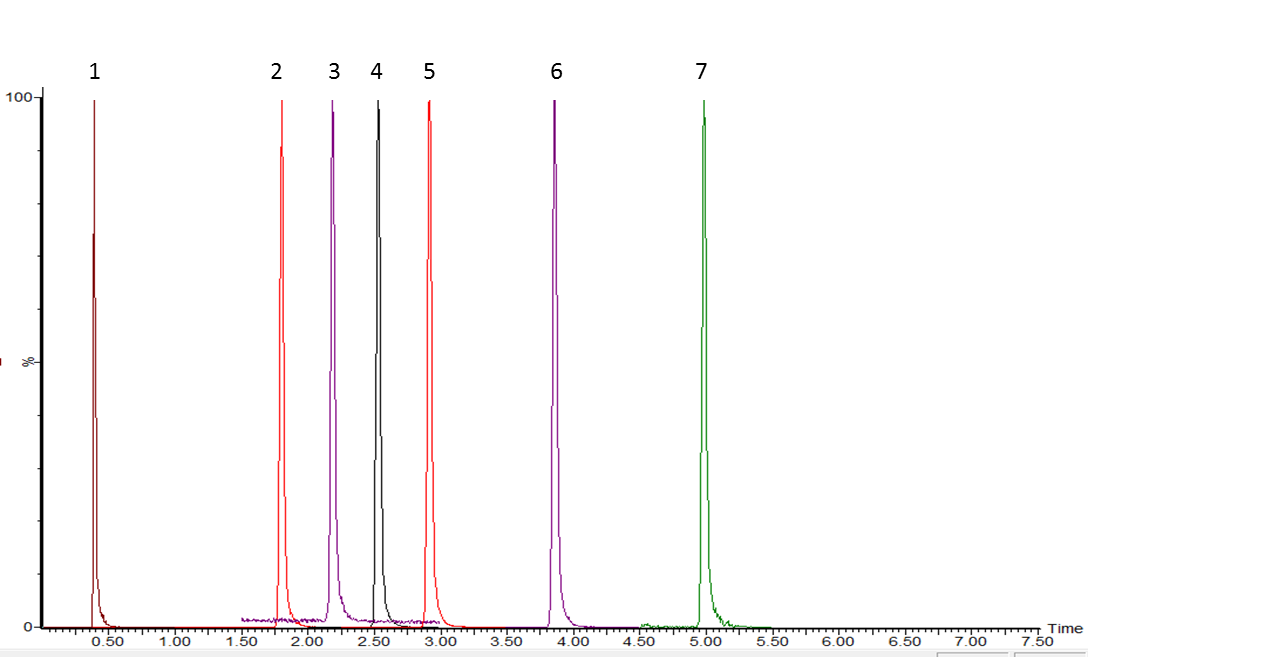


**Figure S12.** Chromatogram showing overlaid peaks for each of the metabolites measured by the method 1=PAP-G, 2= APAP-G, 3= APAP-S, 4= APAP-C, 5= APAP, 6= APAP-GSH, 7=APAP-NAC


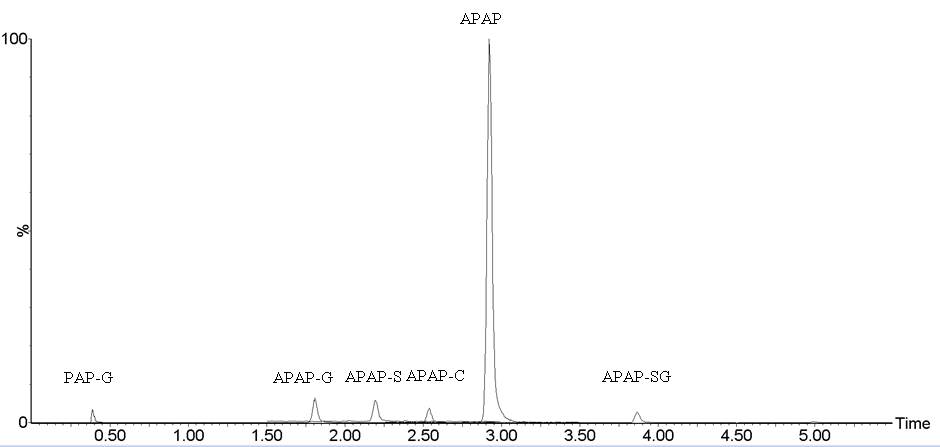


**Figure S13**. Representative total ion chromatogram of APAP metabolites in porcine plasma after 4 hours of dosing.
